# Metabolic profiling of *Escherichia coli* under deuterium oxide-induced stress

**DOI:** 10.1007/s11306-026-02532-3

**Published:** 2026-09-23

**Authors:** Sahand Shams, Ivayla Roberts, Ahmed Nusaim Satheek Rehuman, Nigel Gotts, Yun Xu, Shwan Ahmed, Fabian Jourdan, Catherine Winder, Adam Burke, Royston Goodacre, Howbeer Muhamadali

**Affiliations:** 1 Department of Biochemistry, Cell and Systems Biology, Centre for Metabolomics Research, Institute of Systems, Molecular and Integrative Biology, University of Liverpool, Liverpool, UK; 2 Department of Environment and Quality Control, Kurdistan Institution for Strategic Studies and Scientific Research, Kurdistan Region, Iraq; 3 Toxalim (Research Centre in Food Toxicology, Université de Toulouse, INRAE, ENVT, INP-Purpan, UPS, Toulouse, France

**Keywords:** Metabolic profiling, E. coli, Stable isotope probing, Deuterium oxide

## Abstract

**Introduction:**

Some bacteria tolerate deuterium oxide (D_2_O) and are able to grow efficiently, while others have slower growth phenotypes. Thus, for some bacteria D_2_O perturbs hydrogen-dependent enzymatic processes through kinetic isotope effects, yet the resulting metabolic adaptations in bacteria are not fully understood. In particular, the biochemical factors underlying strain-specific tolerance to D_2_O remain unclear.

**Objectives:**

This study aimed to characterise the global metabolic responses of D_2_O-susceptible and D_2_O-tolerant *Escherichia coli* isolates, and to identify pathways and metabolites associated with isotopic stress tolerance.

**Methods:**

Five uropathogenic *E. coli* isolates and the reference strain *E. coli* K-12 MG1655, were grown in minimal medium supplemented with glucose and casamino acids containing 0%, 40%, and 80% D_2_O. Growth profiles were monitored, and intracellular metabolites were extracted at strain- and condition-specific time points corresponding approximately to the midpoint of the maximum observed OD₆₀₀ . Untargeted LC-MS analysis was performed in both positive (ESI+) and negative (ESI-) ionisation modes, and data were processed using established metabolomics workflows. Statistical analyses included PCA, PC-DFA, two-way ANOVA (FDR < 0.05), fold-change assessment, and pathway enrichment analysis.

**Results:**

Growth profiling showed strain-dependent and progressively greater inhibition with increasing D_2_O concentration, with stronger effects in the susceptible isolates. Metabolomic analysis identified 44 significantly altered metabolites across nine metabolic pathways, including central carbon metabolism, amino acid degradation, nucleotide metabolism, and redox processes. Susceptible strains showed pronounced changes in metabolites associated with glutathione and polyamine metabolism, nicotinate and nicotinamide metabolism, nucleotide metabolism and central-carbon-associated processes. In contrast, tolerant strains generally showed more moderate abundance changes in several of these metabolite groups. Additional responses in susceptible isolates included changes in amino acid-, dipeptide- and polyamine-associated metabolites, whereas tolerant isolates generally showed more moderate responses.

**Conclusions:**

D_2_O exposure was associated with significant abundance changes in metabolites involved in central carbon-associated, amino acid, nucleotide, nicotinate and nicotinamide, glutathione and polyamine metabolism in *E. coli*. The metabolic trajectories of susceptible *versus* tolerant isolates diverge significantly, revealing strain-specific adaptive strategies. These findings provide a biochemical basis for isotopic stress tolerance and enhance our understanding of how bacteria remodel metabolism under heavy-water exposure.

**Supplementary Information:**

The online version contains supplementary material available at https://doi.org/10.1007/s11306-026-02532-3.

## Introduction

Microbial ecologists have long aimed to link the phylogeny of (currently) uncultured microorganisms with their functional roles in complex ecosystems. Advances in multi-omics technologies, combined with stable isotope probing (SIP), now enable direct connections between microorganisms and their metabolic activities, offering new insights into how diverse taxa contribute to environmental processes. SIP has become a key approach for connecting microbial identity with function (Manefield et al., 2002). SIP uses non-radioactive isotopes of elements such as deuterium (^2^H or D), ^13^C, ^15^N, and ^18^O to trace biochemical processes, based on the principle that an organism’s isotopic composition reflects the substrates it assimilates. This method allows researchers to follow resource utilisation and activity *in situ*. Deuterium from D_2_O is a particularly powerful SIP label because water participates in nearly all biochemical reactions (Kobak et al., 2022). As a universal tracer, D_2_O has been widely applied in microbiology and metabolomics to monitor biosynthesis, pathway activity, and metabolic fluxes by tracking deuterium incorporation into cellular molecules (Berry et al., 2015; Opitz et al., 2019; Shams et al., 2023, 2025a, b; Wang et al., 2020, 2024). However, the substitution of hydrogen with deuterium strengthens chemical bonds and lowers zero-point vibrational energies, resulting in less flexible bonds (Kuchitsu, & Bartell, 1962). These physicochemical changes give rise to primary and secondary kinetic isotope effects (KIEs) (Hammes-Schiffer, 2025), which slow hydrogen-dependent processes such as proton-coupled electron transfer and hydride transfer (Layfield, & Hammes-Schiffer, 2014). Consequently, deuterium incorporation disrupts reaction kinetics and equilibria, leading to metabolic imbalances that can alter pathway fluxes and impair cellular growth and overall metabolic activity (Opitz et al., 2019).

While high concentrations of D_2_O are known to impair growth in plants and animals (Flaumenhaft et al., 1965), some microorganisms, including bacteria, algae, yeast, and certain unicellular eukaryotes, can tolerate substantial levels of heavy water. Even so, these organisms experience slowed growth and metabolic adjustments when exposed to D_2_O, indicating that deuteration imposes physiological stress even in relatively tolerant species (Flaumenhaft et al., 1965; Unno et al., 1988).

Although D_2_O-induced KIEs have been quantified for individual enzymes *in vitro*, system-wide consequences have only recently been probed. Hochuli used fractional ^13^C-labelling to show that central carbon fluxes are rerouted towards anaplerosis in 70% D_2_O, implicating TCA cycle inhibition in *E. coli* (Hochuli et al., 2000). More recently, an extensive proteomic study revealed down-regulation of translational machinery and up-regulation of redox-active enzymes in *E. coli* grown on fully deuterated medium (Opitz et al., 2019). Yet proteome data alone cannot capture the small-molecule layer where KIEs are first expressed.

Untargeted metabolomics enables broad detection of metabolites, including labile cofactors and pathway intermediates that often reveal changes occurring before transcriptional or translational regulation. By capturing global shifts in small-molecule composition, this approach is well-suited for identifying metabolic responses to environmental stressors (Hollywood et al., 2006).

Abilev showed that D_2_O induces DNA damage responses, including *recA* activation and coordinated stress-response gene expression (Abilev et al., 2023). Although these studies highlight major physiological impacts, they focus primarily on proteins and gene regulation. The metabolite-level consequences of D_2_O stress remain poorly characterised, even though KIEs manifest first at the chemical and metabolic level, underscoring the need for untargeted metabolomics to resolve these early perturbations.

In this study, we characterise the global metabolic response of *E. coli* to D_2_O stress using untargeted liquid chromatography-mass spectrometry (LC-MS)-based metabolomics. By integrating high-resolution MS with systematic analyses, we identified metabolite-level perturbations induced by D_2_O exposure and implied associated shifts in metabolic pathway activity. These results complement existing proteomic data and provide insight into the biochemical mechanisms underlying cellular adaptation to isotopic stress. A deeper understanding of these metabolic responses has implications for metabolic engineering, the development of deuterated compounds, and the refinement of SIP methodologies in microbiology.

## Materials and methods

### Chemicals, microorganisms, and growth conditions

 Unless otherwise specified, all reagents were sourced from Sigma-Aldrich (UK). The model strain *Escherichia coli* K-12 MG1655, along with a subset of uropathogenic *E. coli* (UPEC) isolates selected from (Dawson, 2003), were revived from − 80 °C glycerol stocks and streaked onto Luria-Bertani (LB) agar plates to obtain single colonies. These isolates underwent three consecutive sub-cultures on LB agar at 37 °C overnight to ensure culture purity before being inoculated into LB broth. For subsequent growth in minimal medium, single colonies were used to inoculate 50 mL of M9 minimal medium (Table S1) supplemented with 5 g/L glucose and 5 g/L casamino acids (MMCAA) in 250 mL Erlenmeyer flasks. Cultures were incubated at 37 °C with shaking at 180 rpm for 18 h in a Brunswick G25 shaker incubator (GMI, USA).

### Growth profiling of UPEC isolates in deuterated media

To assess bacterial tolerance to D_2_O, cultures were prepared in MMCAA adjusted to contain varying D_2_O percentage (0%, 20%, 40%, 60%, and 80%) in H_2_O. Following the initial culture conditions, bacterial suspensions were standardised to an optical density of 0.1 at 600 nm (OD₆₀₀) using a spectrophotometer. Data points represent the mean OD₆₀₀ of three technical replicate wells derived from the same culture, and error bars represent the standard deviation among these wells. Growth profiles were monitored over 24 h using a Bioscreen C automated growth analysis system (Oy Growth Curves Ab Ltd., Finland) under the following settings: preheating for 10 min, continuous shaking, 10 min measurement intervals, and incubation at 37 °C.

### LC-MS analysis

#### Quenching and extraction of intracellular metabolites

Based on the growth profiles, three D_2_O-susceptible and three D_2_O-tolerant strains were selected for metabolomic analysis. The strains were cultured overnight in MMCAA and diluted to an initial OD₆₀₀ of 0.1 in 15 mL of fresh MMCAA containing 0%, 40%, or 80% D_2_O. Cultures were incubated at 37 °C with shaking at 180 rpm. Because D_2_O altered the growth profiles differently among strains, a single fixed collection time was not used. For each strain and D_2_O condition, the target sampling point was selected at approximately the midpoint of the observed OD₆₀₀ increase, i.e. approximately halfway between the initial OD₆₀₀ and the maximum OD₆₀₀ observed in the corresponding growth curve, rather than halfway through the incubation time. Where strains or conditions showed similar growth profiles, common collection times were selected where possible to facilitate experimental sampling while maintaining approximately comparable relative positions within their respective growth curves. All cultures grown at 0% D_2_O were collected after 3 h. At 40% D_2_O, MG1655 and strains 25 and 148 were collected after 3 h, whereas strains 99, 133 and 134 were collected after 7 h. At 80% D_2_O, MG1655 and strains 25 and 148 were collected after 4.5 h, strain 99 after 9 h, and strains 133 and 134 after 10 h. This approach was used to collect cultures at broadly comparable positions within their respective exponential-growth profiles rather than at the same chronological time. For each condition, five biological replicates were collected. To arrest metabolism, the remaining culture volume (15 mL) was immediately added to 30 mL of −48 °C pre-chilled 60% methanol and mixed rapidly. This quenching step arrested intracellular reactions without causing metabolite leakage (Winder et al., 2008). Samples were centrifuged at 4347 rcf for 10 min at −8 °C to harvest the cells, and a brief secondary spin was performed to remove residual supernatant. Pellets and supernatants were stored at −80 °C for further processing. For metabolite extraction, frozen pellets were resuspended in 500 µL of 80% methanol (pre-chilled to −48 °C) and transferred to 2 mL tubes. An additional 500 µL of the same solvent was used to rinse the original tubes, ensuring full biomass recovery. Samples were thawed on wet ice and vortexed vigorously. Three cycles of freezing in liquid nitrogen followed by thawing and vortexing were performed to lyse the cells and enhance metabolite recovery (Muhamadali et al., 2015). The lysates were then centrifuged at 13,000 rcf for 5 min at − 9 °C, and the resulting supernatants were transferred to fresh tubes and maintained on wet ice. The OD₆₀₀ measured immediately before harvest was used as a proxy for the amount of biomass extracted. To normalise the extracts, the lowest harvest OD₆₀₀ measured across the experimental samples was used as the reference. Extracts from samples with higher harvest OD₆₀₀ values were diluted with 80% methanol to the same OD₆₀₀-equivalent concentration using the relationship C₁V₁ = C_2_V_2_, where C₁ was the harvest OD₆₀₀ of the sample and C_2_ was the lowest harvest OD₆₀₀ used as the normalisation target. The resulting normalised extracts were subsequently dried and analysed by LC-MS. Quality-control (QC) samples were generated by pooling 50 µL from each normalised extract, thoroughly mixing the pooled material, and dividing it into four aliquots of 1,125 µL each (Dunn et al., 2011). Finally, all samples were dried using a vacuum concentrator (Eppendorf 5301, UK) at 35 °C for 14 h and stored at − 80 °C until further analysis.

#### LC-MS setup and data pre-processing

Untargeted metabolomics data were acquired using a Thermo Fisher Scientific Vanquish ultra-high-performance liquid chromatography (UHPLC) system coupled to a Q Exactive Orbitrap mass spectrometer (Thermo Fisher Scientific, UK). Chromatographic separation was performed on a Hypersil GOLD aQ C18 column (100 × 2.1 mm, 1.9 μm particle size; Thermo Fisher Scientific, UK) maintained at 50 °C. The mobile phases consisted of water (solvent A) and methanol (solvent B), both containing 0.1% formic acid. The chromatographic gradient was run for 15 min at a flow rate of 0.4 mL/min, starting at 1% B and progressing through the following steps: 5% B at 1 min, 15% B at 2 min, 50% B at 3 min, and 99% B at 8 min. This composition was held until 11 min, after which it was returned to 1% B by 11.5 min. Samples were maintained at 4 °C in SureSTART WebSeal 96-well plates (Thermo Fisher Scientific, UK), and a 2 µL injection volume was used for analysis.

The mass spectrometer was operated separately in both ESI + and ESI- modes. Spray voltage was set to 3.2 kV (ESI+) and − 2.8 kV (ESI-), with a capillary temperature of 263 °C and auxiliary gas heater temperature of 425 °C. Sheath gas, auxiliary gas, and sweep gas flows were set to 50, 12, and 2 arbitrary units, respectively. The S-lens RF level was adjusted to 58.5%. Full MS scans were acquired in the Orbitrap mass analyser across an *m/z* range of 66.7 to 1000 at a resolving power of 70,000 (FWHM at *m/z* 200). The chromatographic peak width was set to 6 s (FWHM), with an automatic gain control (AGC) target of 1 × 10⁶ and a maximum injection time of 100 ms.

Data-dependent MS/MS (ddMS²) acquisition was performed on pooled QC samples at the end of the analytical batch, following the protocol described by (Broadhurst et al., 2018). Consistent with methods reported by (Mullard et al., 2015), ddMS² data were collected for four precursor *m/*z ranges in triplicate injections: 66.7–1000, 66.7–300, 300–600, and 600–900. MS/MS data were acquired at a resolving power of 17,500 (FWHM at *m/z* 200), with an AGC target of 1 × 10^5^, maximum injection time of 54 ms, loop count of 5, and isolation window of 1.2 *m/z*. Stepped normalised collision energies (NCE) of 20, 50, and 80 were applied. Additional settings included exclusion of isotopes, minimum AGC target of 1 × 10^3^, and dynamic exclusion set to 6 s.

Raw LC-MS/MS data were processed using Compound Discoverer 3.3 (Thermo Fisher Scientific), employing a stable isotope labelling workflow optimised for deuterium-based SIP analysis. Key processing steps included retention time alignment (ChromAlign), compound detection using the Legacy node (minimum intensity: 10,000; mass tolerance: 5 ppm), and compound grouping (retention time tolerance: 0.2 min; peak filtering: 4.5, present in at least 2 files). Background compounds were identified and excluded if their signal intensity in samples did not exceed that in blank injections by at least fivefold. The “Analyse Labelled Compounds” node was used to detect deuterium incorporation, with the labelled element set to [^2^H], maximum exchange set to 25, and source efficiency to 100%. Putative compound identifications were made based on accurate mass and MS/MS fragmentation spectra. Annotations were cross-referenced against mzCloud (Thermo Fisher Scientific), ChemSpider, the Human Metabolome Database (HMDB), the Metabolika pathway database, and a manually curated in-house spectral library. A minimum spectral match score of 70% was applied as the identification threshold. Annotation criteria followed the guidelines defined by (Summer et al., 2007). Final processed datasets were exported in Microsoft Excel (.xlsx) format for downstream statistical and comparative analyses. This workflow enabled high-confidence detection of metabolites, quantification of isotopologue distributions, and characterisation of metabolic shifts associated with D_2_O exposure.

##### Data processing and statistical analysis

Following raw data deconvolution, feature area values were exported as CSV files for further analysis. Data quality assessment was performed manually using criteria adapted from (Dunn et al., 2012). Three key filtering thresholds were applied to ensure the reliability of the data. First, a detection rate threshold of 75% was used, meaning that any metabolite features detected in fewer than 75% of its occurrences across the QC features was excluded. Second, the relative standard deviation (RSD) was calculated for each feature within QC replicates, and those with an RSD greater than 30% were removed due to poor reproducibility. Third, features showing significant background interference were excluded if the average signal in blank samples exceeded 10% of the average signal in QC samples, indicating potential contamination.

After quality filtering, the processed datasets from both ESI + and ESI- modes were uploaded to MetaboAnalyst. Gap filling was performed using the *k*-nearest neighbours (KNN) algorithm as suggested by (Gromski et al., 2014), followed by signal drift and batch effect correction using QC-based robust LOESS signal correction (QC-RLSC) (Dunn et al., 2011). The corrected datasets were then downloaded for further statistical analysis. Univariate and multivariate analyses were carried out using MATLAB version R2023a (code is available on Github: https://github.com/Biospec/). All identified metabolites were treated as Level 2 annotations according to the metabolomics standards initiative (MSI); (Summer et al., 2007), based on accurate mass and MS/MS fragmentation data. Annotations were verified against mzCloud (Thermo Fisher Scientific), the Human Metabolome Database (HMDB), and an in-house spectral library, with a minimum spectral match score of 70% used as the cut-off for putative identification.

Prior to univariate statistical analysis, the data were log₁₀-transformed to reduce skewness. For multivariate statistical analysis, in addition to log₁₀ transformation, sum normalisation and Pareto scaling were applied. Principal component analysis (PCA) (Abdi & Williams, 2010; Wold et al., 1987) was conducted to assess data quality using QC samples. Principal component–discriminant function analysis (PC-DFA) was employed to enhance group separation and identify metabolic patterns associated with D_2_O exposure and strain phenotype. By applying discriminant analysis to the principal component space, PC-DFA increases the interpretability of complex datasets and maximises between-group variance while minimising within-group variance (Goodacre et al., 1998; Gromski et al., 2015). This approach enabled clearer visualisation of clustering among experimental groups and facilitated the identification of key metabolites that contribute most significantly to the observed group separation.

Two-way analysis of variance (ANOVA) was employed to evaluate the effects of both D_2_O concentration and strain phenotype (susceptible *vs*. tolerant) on metabolite abundance. Statistical significance was determined using a Benjamini-Hochberg (BH) false discovery rate (FDR)-adjusted *p*-value (Benjamini, & Hochberg, 1995) threshold of 0.05. Fold-change analysis was additionally performed separately within the susceptible and tolerant phenotype groups to assess the direction and magnitude of metabolite abundance changes across consecutive D_2_O concentrations. Within each phenotype, metabolite abundances were compared between 0% and 40% D_2_O and between 40% and 80% D_2_O. A fold change of 1 indicated no change, values below 1 indicated decreased abundance, and values above 1 indicated increased abundance. Fold-change results were used to support the interpretation of directional changes in metabolite abundance. All analyses were performed separately for ESI + and ESI− ionisation modes. Finally, pathway enrichment analysis was performed using MetaboAnalyst to provide a systems-level overview of pathway-associated metabolic perturbations under D_2_O exposure. To assess whether phenotype-level pooling obscured within-phenotype strain variation, an additional strain-level sensitivity analysis was performed separately for susceptible and tolerant isolates at 0%, 40% and 80% D_2_O using R version 4.6.1 (R Core Team, Austria). For each evaluated feature, linear mixed-effects models were fitted to log₁₀-transformed abundance data, with strain identity included as a fixed effect and experimental replicate included as a random intercept. A model containing the strain effect was compared with an intercept-only model containing the same random-effects structure using a likelihood-ratio test. The resulting *p*-values were adjusted across features within each phenotype, D_2_O concentration and ionisation-mode block using the BH FDR procedure, with an adjusted *p*-value below 0.05 considered significant. Analyses were performed independently for ESI + and ESI- modes data.

In addition to pathway enrichment analysis, network-based metabolic analysis was performed using the *E. coli* K-12 MG1655 metabolic network derived from the KEGG database. The network was downloaded on February 20th, 2026, *via* the MetExplore web server (biosource ID: 7767), comprising 1335 metabolites, 1573 reactions, and 959 genes. All analyses were conducted using the MetExplore web server (version 2) (Cottret et al., 2018). A total of 38 metabolites identified in this study were successfully annotated with KEGG identifiers, of which 35 were mapped to at least one reaction within the metabolic network. These mapped metabolites were distributed across 92 of the 105 metabolic pathways represented in the network.

To identify significantly enriched pathways, over-representation analysis (ORA) was performed using a hypergeometric test (Wieder et al., 2021), followed by Benjamini-Hochberg FDR correction. A total of 30 pathways showed significant enrichment (corrected *p*-value < 0.05). It is important to note that pathway enrichment results should be interpreted with caution, as a defined background metabolite set was not available. This limitation arises from the incomplete coverage inherent to untargeted metabolomics, where the full set of detectable metabolites is unknown. Potential biases and limitations of ORA in metabolomics data have been discussed previously (Cooke et al., 2025; Wieder et al., 2021). 

All pathway enrichment results were provided in Supplementary Information section Table S3. The pathway panel summarises enrichment results, while the metabolite panel listed all annotated metabolites and indicates their statistical significance and mapping status. To further investigate central metabolic perturbations, the carbon metabolism pathway was selected for detailed analysis (FDR adjusted *p*-value = 0.047; 102 reactions, 104 metabolites). A sub-network was constructed by retaining only reactions belonging to this pathway and removing ubiquitous side compounds (e.g., H_2_O, O_2_, ATP, ADP).

## Results and discussion

### Growth profile of UPEC isolates in deuterated media

**Fig. 1:**
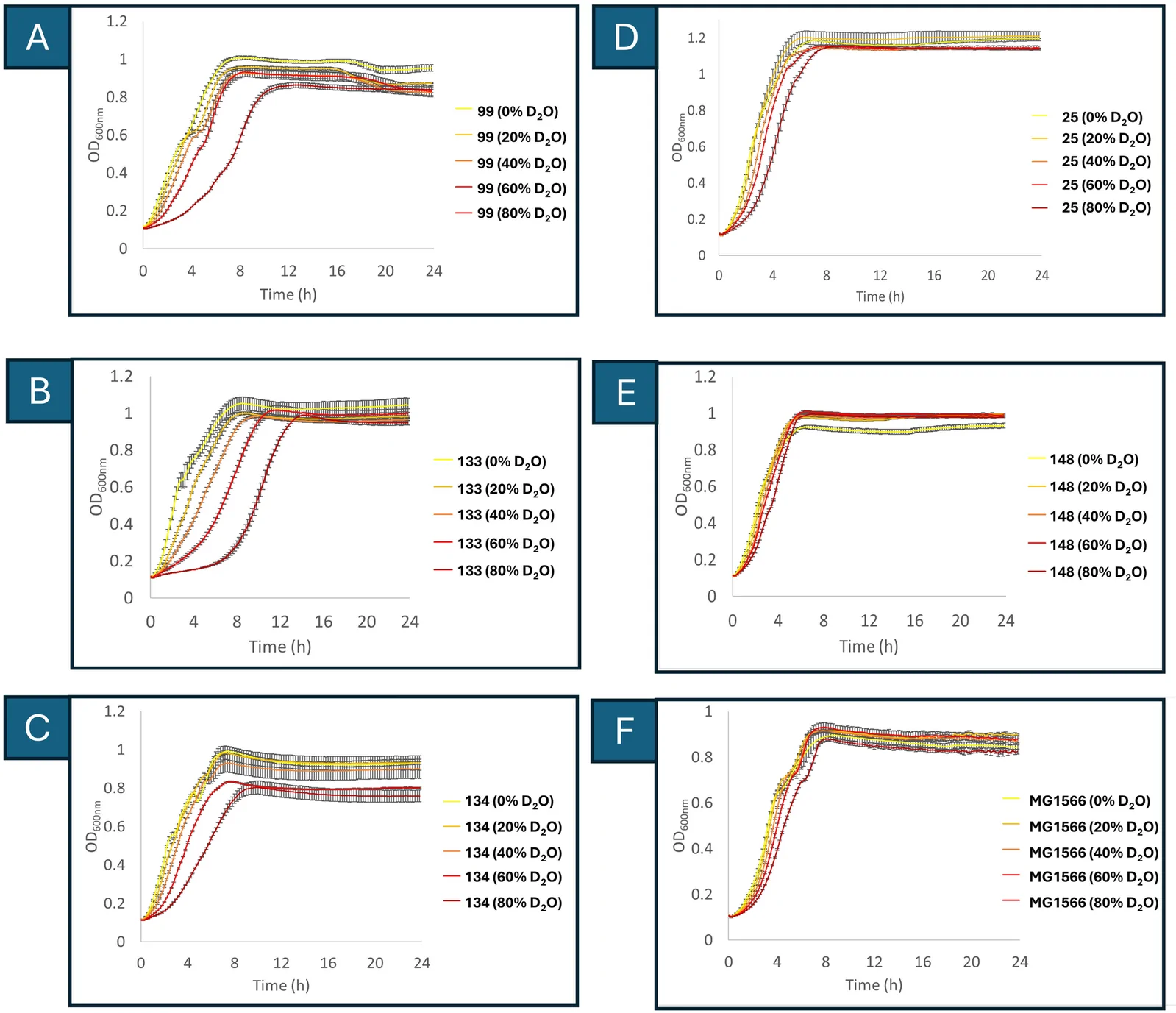
Growth profiles of six *E. coli* strains, comprising five UPEC isolates and the reference strain K-12 MG1655, cultured in deuterated MMCAA with increasing concentrations of D_2_O (0%, 20%, 40%, 60%, and 80%). Panels A-C show growth profiles of isolates 99, 133 and 134, which displayed greater D_2_O sensitivity, characterised by progressively delayed increases in OD₆₀₀ and, for isolates 99 and 134, reduced final OD₆₀₀ values at higher D_2_O concentrations. Panels D-F show isolates 25 and 148 and the reference strain MG1655, which displayed smaller changes in their growth profiles and final OD₆₀₀ values under increasing D_2_O exposure. Points show means while error bars represent standard deviations of biological replicates (*n* = 3). Different colours represent different D_2_O concentrations

To evaluate the impact of D_2_O on bacterial growth, the six selected *E. coli* strains were cultured in MMCAA containing 0%, 20%, 40%, 60% or 80% D_2_O. As shown in Fig. 1, the strains exhibited two broader patterns of response. Isolates 99, 133 and 134 (Fig. 1-A to C) showed greater sensitivity to increasing D_2_O concentrations. Their increases in OD₆₀₀ became progressively delayed and, in some strains, more gradual as D_2_O concentration increased. Changes in the growth profile were already apparent at 20% D_2_O in some isolates, particularly isolate 133, with more pronounced effects at higher concentrations. The growth profiles were therefore interpreted as showing gradual, strain-dependent inhibition rather than a single critical D_2_O tipping point. Previous studies have similarly reported concentration-dependent inhibition of bacterial growth under D_2_O exposure (Berry et al., 2015; Flaumenhaft et al., 1965; Katz & Crespi, 1966; Unno et al., 1988). In contrast, isolates 148 and 25 and the reference strain MG1655 (Fig. 1-D to F) showed smaller changes in their OD₆₀₀ profiles across increasing D_2_O concentrations. Their increases in OD₆₀₀ occurred earlier than those of the susceptible isolates under 40% and 80% D_2_O, and the overall changes in their growth profiles were less pronounced. At 80% D_2_O, the increase in OD₆₀₀ was delayed and more gradual, but this effect remained less pronounced than in isolates 99, 133 and 134. The growth experiments were designed to assess relative D_2_O sensitivity, support strain classification and guide strain- and condition-specific metabolomic sampling, rather than to provide model-based estimates of maximum specific growth rate, lag duration or final growth yield. These kinetic parameters were therefore not formally estimated or statistically compared.

### Metabolic profiling by LC-MS

**Fig. 2:**
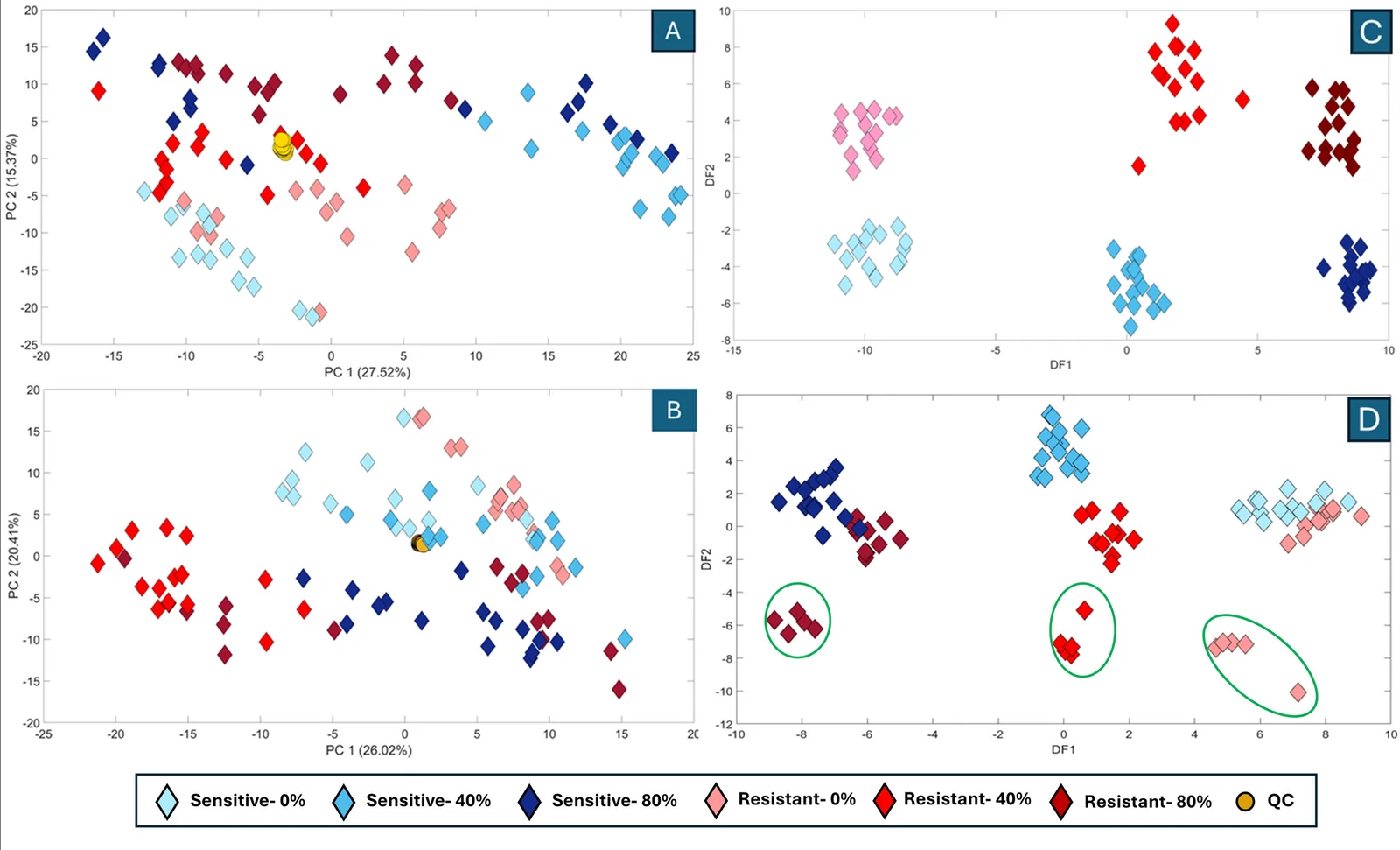
PCA scores plots for ESI+ (TEV: 42.89%) (**A**) and ESI- (TEV: 46.43%) (**B**) and PC-DFA score plots for ESI+ (**C**) and ESI- (**D**), respectively, performed using supervised classification with all biological replicates of each condition defined as a class LC–MS data from UPEC isolates grown in varying concentrations of D_2_O. UPEC isolates 99, 133 and 134 were classified as D_2_O-susceptible, whereas UPEC isolates 25 and 148 and the K-12 strain MG1655 showed the relatively D_2_O-tolerant phenotype. In all plots, colours denote D_2_O tolerance phenotypes, with blue tones indicating susceptible isolates and red tones indicating tolerant isolates; increasing D_2_O concentration is indicated by progressively darker shades. Yellow symbols represent pooled QCs. Green circles in panel **D** highlight the *E. coli* MG1655 strain

LC-MS metabolomic analysis was performed on *E. coli* MG1655 and five UPEC isolates using the UHPLC-Q Exactive Orbitrap method described in Section. 2.3.2, with data acquired independently in + and ESI-. Initially, 7,008 metabolite features were detected in ESI+, including 195 putatively annotated metabolites, and 2,800 metabolite features were detected in ESI−, including 52 putatively annotated metabolites. Following data preprocessing, including deconvolution, blank filtering and QC filtering, 1,864 features, including 60 putatively annotated metabolites, remained in ESI+, while 1,337 features, including 37 putatively annotated metabolites, remained in ESI−. Putative metabolite annotations corresponded to MSI Level 2 assignments based on accurate mass and MS/MS spectral matching against reference databases and the in-house spectral library, using a minimum spectral match score of 70%. Because authentic chemical standards were not used for confirmation, these annotations were not considered MSI Level 1 identifications. The filtered features were retained for subsequent statistical analysis. PCA score plots of all samples in both ESI+ (Fig. 2-A) and ESI- (Fig. 2-B) , including both susceptible and tolerant isolates under all D_2_O concentrations (0%, 40%, and 80%), showed tight clustering of the pooled QC samples around the centre (yellow circle symbols in Fig. 2-A and B), indicating minimal variation across experimental batches and high reproducibility of these metabolomics data. In the ESI+ (Fig. 2-A), PCA revealed a subtle separation along the PC2 axis; control samples (0% D_2_O) from both susceptible and tolerant isolates were predominantly positioned on the negative side of PC2, whereas samples exposed to 40% and 80% D_2_O were more frequently positioned on the positive side, although substantial overlap and within-group variation were present. The ESI- showed a weaker separation between control and D_2_O-treated groups. Specifically, samples from both susceptible and tolerant isolates grown in 80% D_2_O, together with tolerant isolates grown in 40% D_2_O, tended to occupy the negative side of PC2, although the groups remained broadly dispersed and overlapping. Meanwhile, control samples and susceptible isolates grown in 40% D_2_O clustered on the positive side of the axis. To enhance clustering patterns and generate models focused on metabolites most strongly affected by D_2_O stress in UPEC isolates, PC-DFA models were constructed using the first 10 principal components from each electrospray ionisation mode. Class membership was defined by the combination of three experimental factors: percentage of D_2_O exposure (0%, 40%, and 80%), phenotypic response to D_2_O (susceptible *vs*. tolerant), and individual strain identity, with three independent strains representing each phenotype. This factorial design resulted in 18 distinct classes in total (3 D_2_O levels × 2 phenotypes × 3 strains), which were used to train the PC-DFA models and to identify metabolites contributing most to discrimination between exposure levels and phenotypic groups. Note that as this *a* *priori* grouping was used for DFA, this does not bias the multivariate analyses as no information on grouping, (e.g.) according to share exposures to D_2_O, is provided to the algorithm. One can therefore consider this as a semi-supervised approach.

The PC-DFA scores plot of LC-MS data in ESI+ (Fig. 2-C), in which the first 10 PCs accounted for 72.48% of the total explained variance (TEV), revealed a clear clustering pattern. Along the DF1 axis, a gradient corresponding to D_2_O exposure was observed; control groups (including both tolerant and susceptible isolates) were located on the negative side of DF1, isolates grown in 40% D_2_O clustered around the centre of the axis (near 0, slightly toward the positive side), and those grown in 80% D_2_O were positioned on the far positive end of DF1. As PC-DFA is a supervised method, class structure in this analysis was defined based on both the percentage of D_2_O exposure (0%, 40%, and 80%) and the phenotypic response of the bacterial strains to D_2_O (susceptible *vs*. tolerant). This dual classification allowed for improved resolution of metabolic shifts associated with treatment and strain-specific responses. It is also important to note that the separation captured by DF1 does not reflect the extent of deuterium incorporation into metabolites. During data pre-processing and deconvolution, based on the features of the CD software, the area of each metabolic feature represents the sum of all ions, including both monoisotopic and deuterium-labelled forms at different incorporation levels. Therefore, the separation observed in DF1 primarily reflects metabolic changes induced by D_2_O exposure, rather than isotopic enrichment.

Additionally, both susceptible and tolerant isolates formed distinct clusters under all conditions along the DF2 axis, indicating inherent metabolic differences between the two phenotypes. The visual separation between susceptible and tolerant isolates along DF2 was most evident at 40% D_2_O. This observation refers specifically to the relative separation between the two phenotype groups and should not be interpreted as indicating that 40% D_2_O produced a greater overall metabolic effect than 80% D_2_O. Along DF1, the 80% D_2_O samples showed the greatest displacement from the 0% D_2_O controls, indicating the largest overall D_2_O-associated shift in the PC-DFA scores space. The reduced phenotype separation at 80% D_2_O may be consistent with both phenotype groups moving towards a more broadly constrained metabolic state under the highest D_2_O exposure; however, this remains an interpretation of the metabolite-abundance patterns rather than direct evidence of metabolic suppression. The PC-DFA scores plot of LC-MS data in ESI- (Fig. 2-D), in which the first 10 PCs accounted for 74.82% of the total TEV, displayed a similar clustering pattern, albeit with weaker separation between the susceptible and tolerant isolates. As in the ESI+, DF1 reflected the effect of D_2_O exposure; control groups (both susceptible and tolerant) were positioned on the positive side of DF1, isolates grown in 40% D_2_O clustered near the centre, leaning slightly toward the positive side and closer to the controls, while those grown in 80% D_2_O shifted toward the far negative end of DF1.

A noteworthy observation was the distinct clustering of *E. coli* MG1655 (highlighted by green circles) across all conditions along the DF2 axis, suggesting metabolic differences between this strain and the other UPEC isolates in the ESI- data. Consistent with the ESI+ results, the visual separation between susceptible and tolerant isolates was greater at 40% D_2_O than at 80% D_2_O. This pattern was interpreted descriptively as greater phenotype separation at 40% D_2_O and not as evidence that the overall effect of D_2_O was greater at 40% than at 80%.

To obtain an initial, qualitative overview of features contributing to the separation between susceptible and tolerant isolates across D_2_O concentrations, DF1 loading plots were generated for the LC-MS data (data not shown) separately for each ionisation polarity. These loading plots were used solely as an exploratory guide to indicate which metabolites appeared to have greater influence on class discrimination, rather than as a formal statistical basis for feature selection. The loading values were extracted and ranked to provide an approximate assessment of relative contribution, with particular attention to the position of known metabolites. Definitive identification of significant discriminatory metabolites was based on two-way ANOVA, and the DF1 observations were used only to support the interpretation of the ANOVA results.

 The two-way ANOVA identified 1,418 metabolite features in ESI+, including 38 MSI level 2 annotated metabolites, and 1086 metabolite features in ESI-, including 17 MSI level 2 metabolites, as being significantly altered in response to D_2_O exposure. Notably, 11 metabolites were found to be commonly identified in both ESI + and ESI-. The list of these altered metabolites, along with their associated FDR adjusted *p*-values and *m*/*z* are provided in Table S2. To assess whether phenotype-level pooling obscured strain-specific variation, strain effects were tested separately within each phenotype and D_2_O concentration. Among the MSI Level 2 annotated metabolites used for pathway analysis and biological interpretation, only 20 of 330 mode-specific metabolite-condition tests (6.06%) showed an FDR-significant strain effect, involving nine unique metabolites while 93.94% showed no detectable effect (data not shown). Thus, phenotype-level pooling was supported for most annotated metabolites, although pooled values for metabolites showing significant strain variation were interpreted as average rather than uniform responses of all strains within that phenotype.

**Fig. 3:**
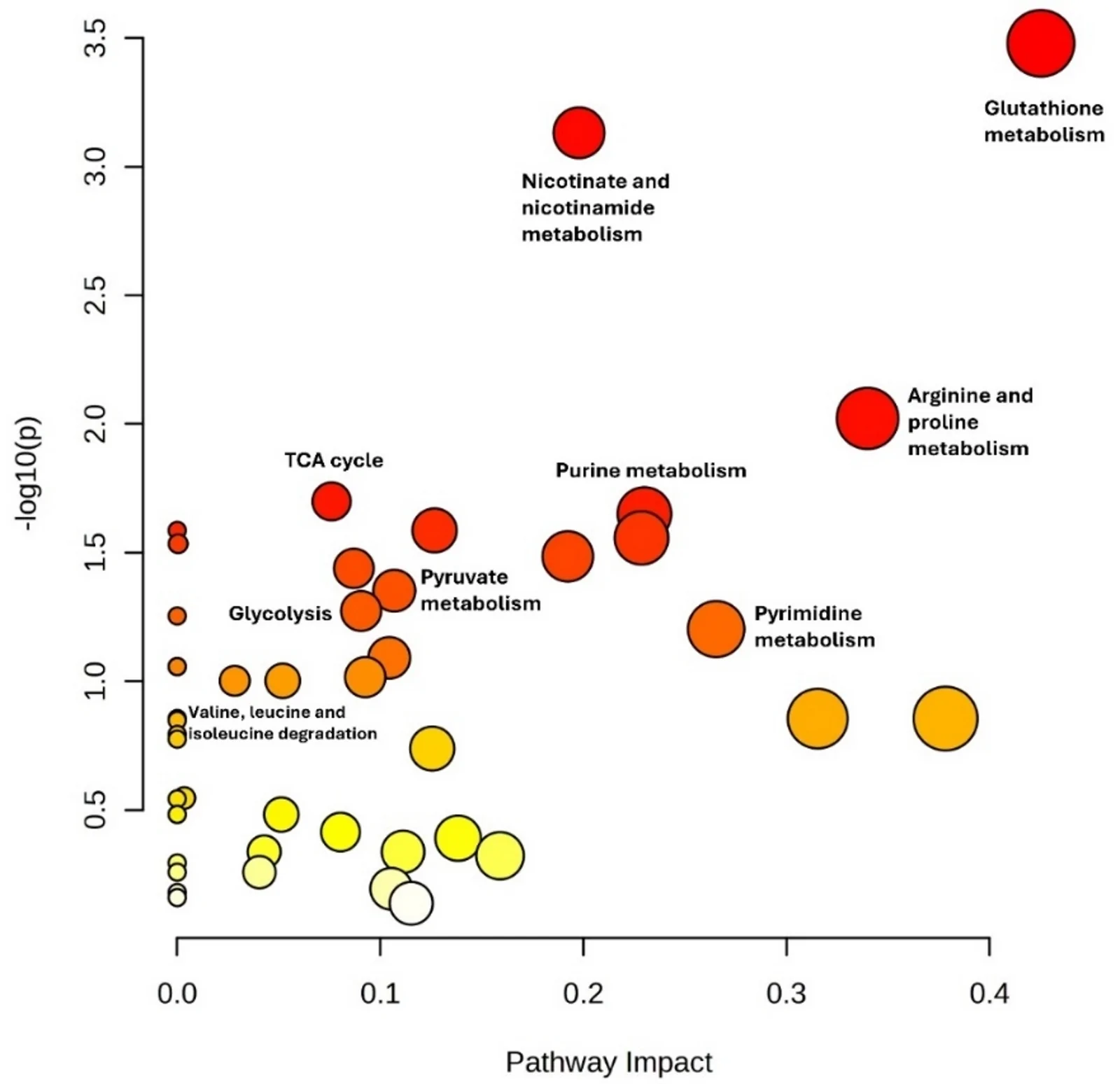
MetaboAnalyst pathway analysis of intracellular metabolites significantly altered under D_2_O exposure in *E. coli*. The x-axis represents pathway impact, a topology-based measure reflecting the relative importance of the matched metabolites according to their positions within the pathway network, while the y-axis represents pathway-enrichment significance as -log₁₀(*p*-value). Larger and darker-coloured circles indicate pathways with greater pathway impact and enrichment significance

Subsequently, pathway enrichment analysis was performed using MetaboAnalyst with the 44 MSI Level 2 annotated metabolites as input to provide a broader overview of metabolic processes associated with D_2_O exposure. For focused biological interpretation, pathway categories represented by at least three significantly altered annotated metabolites were prioritised for further discussion. This threshold was used as an interpretive selection criterion rather than as a statistical definition of pathway activation or suppression. The analysis identified nine pathway categories represented by the detected metabolites, including glutathione metabolism, arginine and proline metabolism, arginine biosynthesis, nicotinate and nicotinamide metabolism, purine and pyrimidine metabolism, glycolysis, pyruvate metabolism, the tricarboxylic acid (TCA) cycle, and valine, leucine and isoleucine degradation (Fig. 3).

 Importantly, pathway membership was not considered mutually exclusive. Individual metabolites, particularly central metabolic intermediates, can participate in several biochemical pathways and therefore may contribute to more than one pathway-enrichment result. The statistical significance and pathway memberships of the 44 MSI Level 2 annotated metabolites are summarised in Table S4, with overlapping pathway assignments indicated where applicable. Accordingly, the assignment of a metabolite to a pathway in this analysis does not imply that the metabolite is specific to that pathway or that changes in its abundance originate exclusively from that metabolic process. Pathway enrichment was used as a systems-level organisational framework for the detected metabolite changes rather than as evidence of pathway-specific activity. Interpretation is additionally limited by the incomplete metabolite coverage inherent to untargeted LC-MS, as several intermediates within these pathways were not detected. The pathway-level results are therefore interpreted as evidence of pathway-associated metabolite perturbations rather than direct evidence of pathway activation, suppression or altered metabolic flux. The database-defined category “biosynthesis of secondary metabolites” was also returned by the pathway enrichment analysis. We recognise that this represents a broad database overview category containing metabolites and reactions shared across multiple biosynthetic and central metabolic routes rather than a single discrete metabolic pathway in *E. coli*. Its enrichment should therefore not be interpreted as evidence for activation or perturbation of a specific secondary-metabolite pathway. The category is retained for completeness in the pathway-enrichment output but is not used for further biological or mechanistic interpretation.

**Fig. 4:**
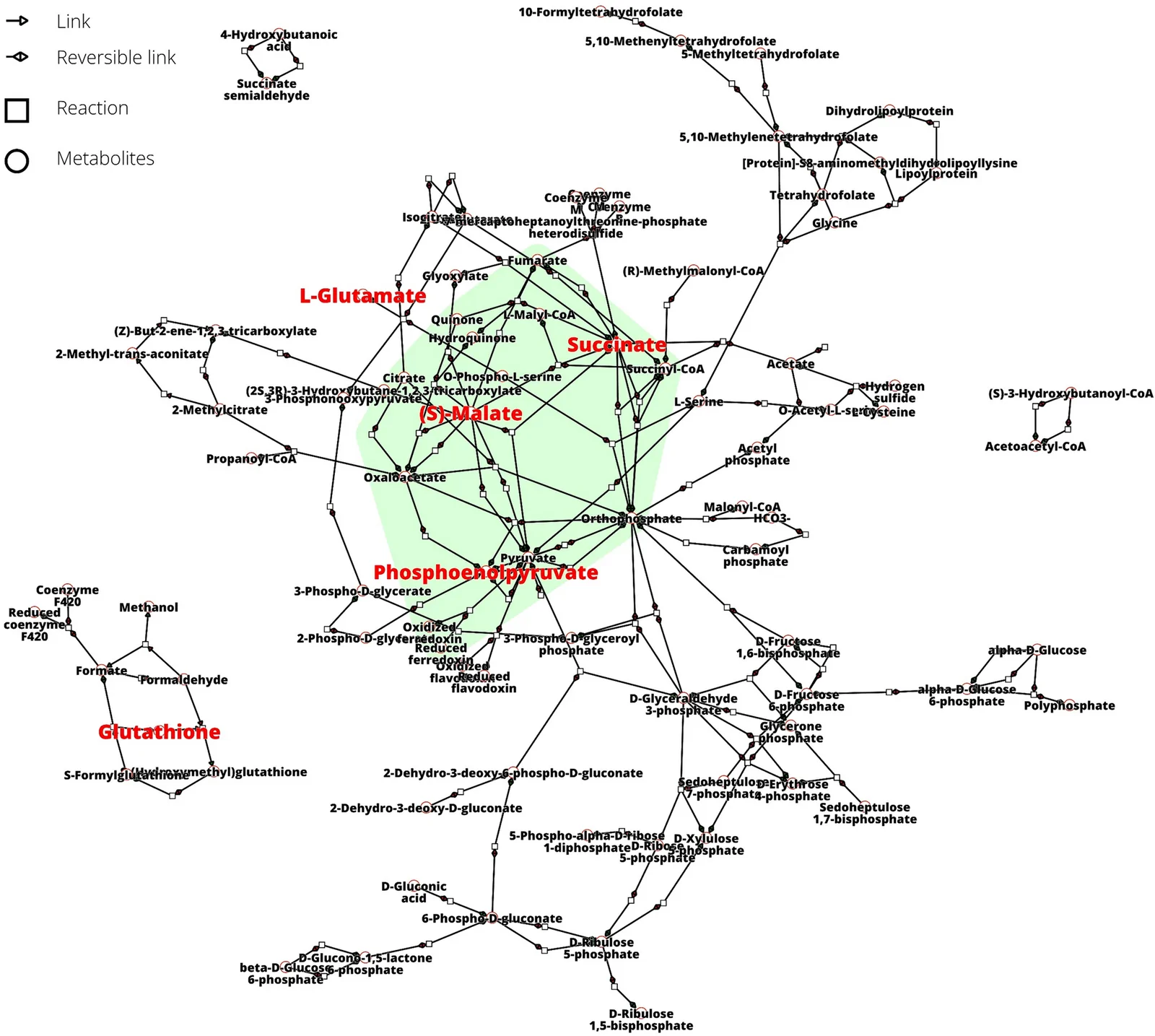
KEGG-based carbon metabolism sub-network generated using MetExplore for *E. coli* K-12 MG1655. The network includes reactions assigned to the Carbon Metabolism pathway after removal of ubiquitous side compounds. Circles represent metabolites and rectangles represent reactions. Metabolites detected in this study are highlighted in red, and edges corresponding to TCA cycle reactions are shown in green. The spatial position of individual nodes reflects the network visualisation layout and should not be interpreted as defining pathway boundaries or pathway membership

To further contextualise pathway-level changes within the global metabolic network, a KEGG-based network analysis was performed using MetExplore. The resulting carbon metabolism sub-network is shown in Fig. 4, highlighting the position of significantly altered metabolites within central metabolic pathways. The network shows that the majority of significantly altered metabolites are localised within central carbon metabolism, including glycolysis and the TCA cycle, as well as amino acid and redox-related pathways, which are explored in detail in the following sections.

**Fig. 5:**
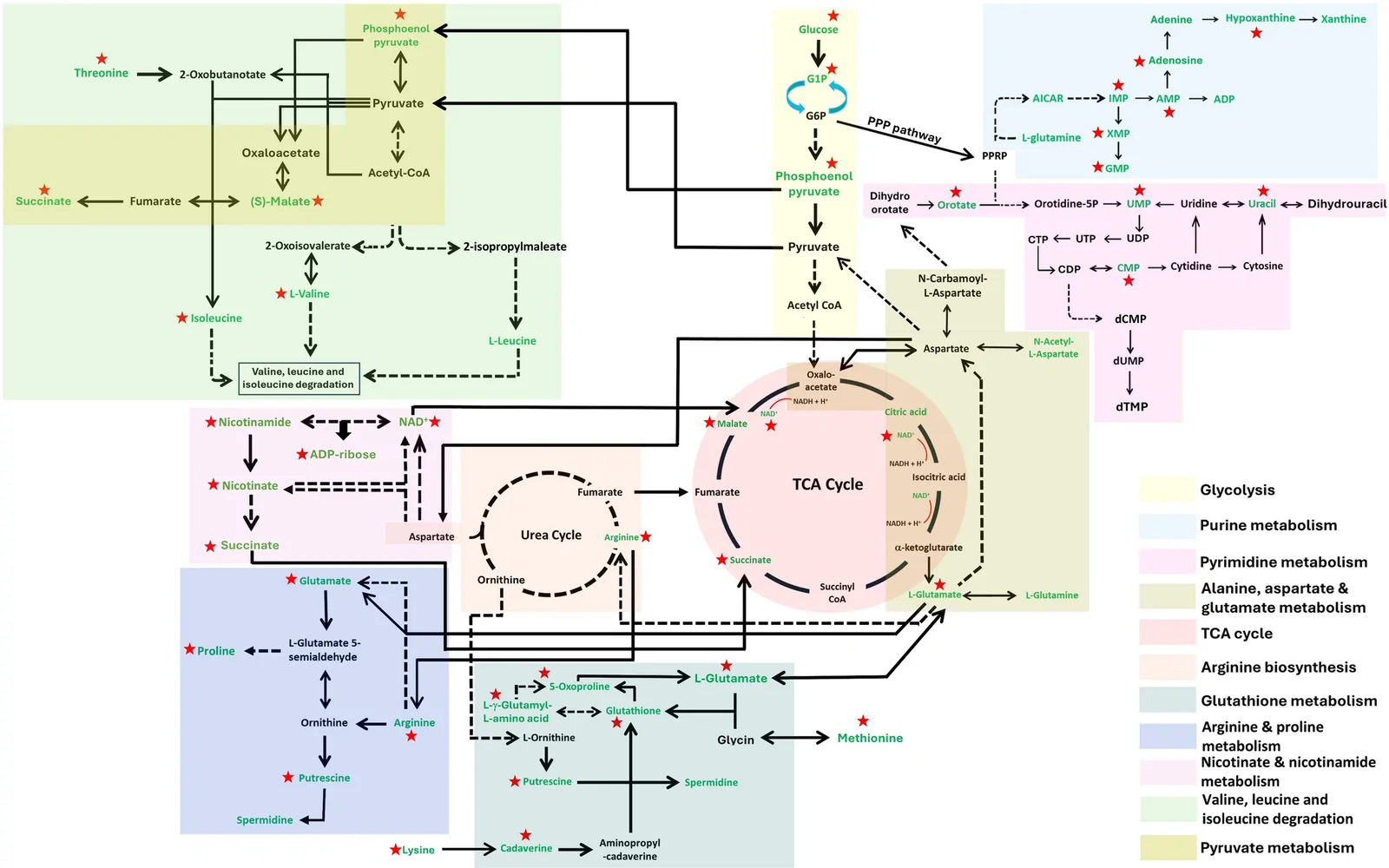
Overview of metabolic pathways affected by D_2_O exposure in *E. coli*, highlighting differences in both tolerant and susceptible isolates. Metabolites identified at MSI level 2 are shown in green, while those showing significant changes (*p* < 0.05) at any D_2_O concentration compared to other concentrations are marked with a red star. Metabolites identified at MSI level 3 or not identified, but were detected, are shown in black. Arrows indicate metabolic connections; solid lines represent direct biochemical links, while dotted lines indicate indirect connections involving intermediate metabolites not shown. Colour shading distinguishes different metabolic pathways. The legend provides the colour codes to assist with the interpretation of the figure.

An overview of the pathway analysis results is presented in Fig. 5, which broadly aligns with findings reported in the literature. For instance, a study by Hochuli and colleagues demonstrated that exposure to D_2_O in *E. coli* primarily affects central carbon metabolism, notably suppressing respiration and the TCA cycle, while leaving glycolysis and the pentose phosphate pathway almost unaffected (Hochuli et al., 2000). This is consistent with our findings, where the most significantly impacted, pathways are generally related to energy production and carbon metabolism, highlighting the notion that D_2_O exposure disrupts core metabolic processes in *E. coli*. Similarly, a study by Opitz showed that D_2_O exposure affects the *E. coli* proteome by altering multiple enzymatic systems, as well as causing a downregulation of translational proteins (Opitz et al., 2019). These proteomic findings provide broader context for the cellular response to D_2_O but are not interpreted here as direct evidence for the specific metabolite changes or pathway-associated patterns observed in the present study.

#### Interpretation of the LC-MS metabolite profile data

**Fig. 6:**
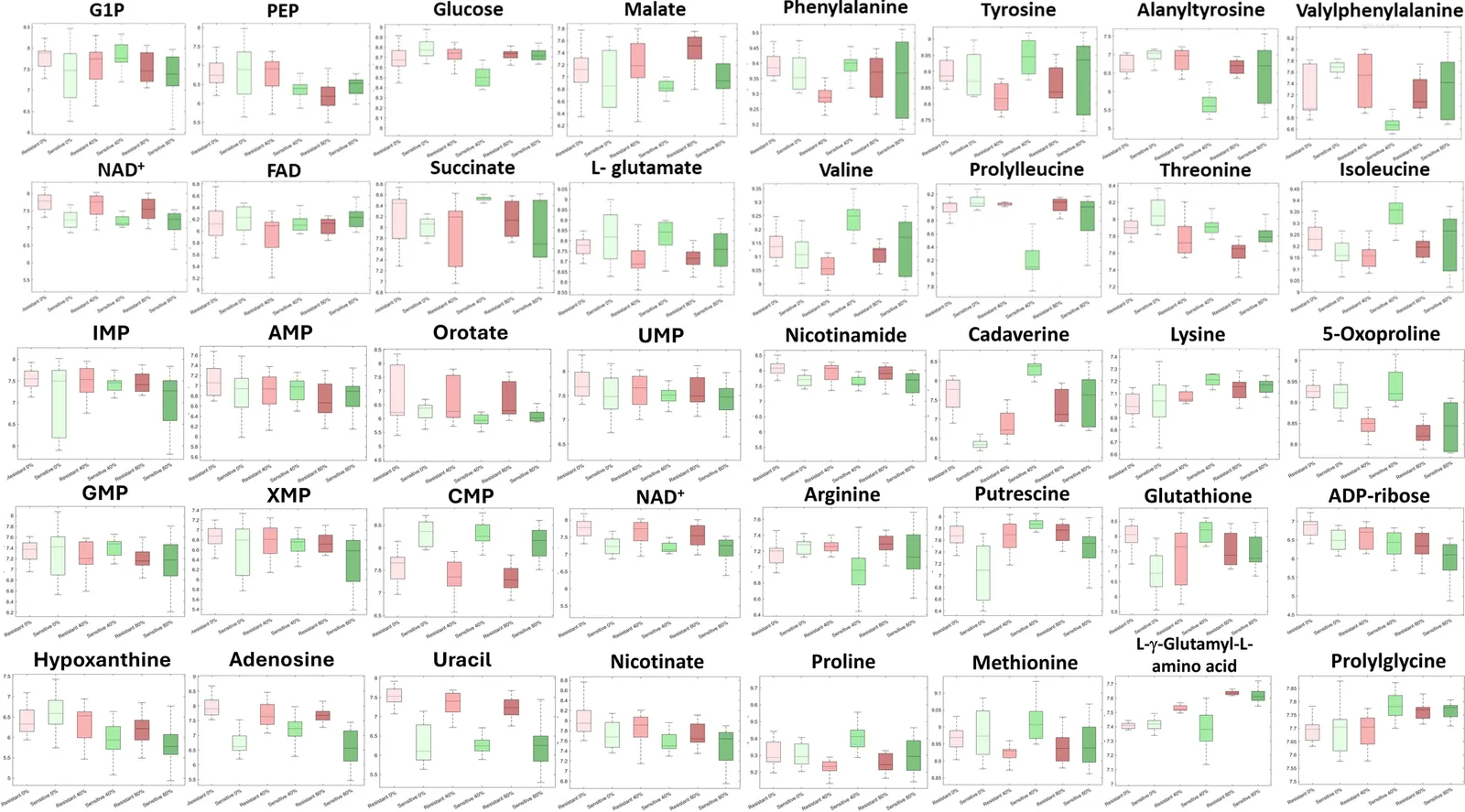
Box-and-whisker plots showing significantly altered metabolites (*p* < 0.05) at any D_2_O changes in all discussed pathways. Box-and-whisker plots showing log₁₀-transformed relative LC-MS intensities of annotated metabolites showing a significant D_2_O effect (BH-FDR adjusted *p* < 0.05) across the discussed pathways. Each set of six boxes is ordered from left to right as: tolerant 0%, susceptible 0%, tolerant 40%, susceptible 40%, tolerant 80%, and susceptible 80% D_2_O. Green indicates susceptible isolates and red indicates tolerant isolates, with darker shades representing higher D_2_O concentrations. Boxes represent the interquartile range, centre lines indicate the median, and whiskers extend to the most extreme values within 1.5 × the interquartile range.

To date, only limited investigations have explored the effects of D_2_O on various *E. coli* strains, with some of the earliest studies dating back to the 1960 s (De Giovanni, 1961). More recently, the impact of D_2_O on *E. coli* has been revisited using multi-omics approaches and advanced analytical tools such as LC-MS (Opitz et al., 2019), GC-MS (Wang et al., 2024), and NMR spectroscopy (Hochuli et al., 2000). However, to the best of our knowledge, no study has specifically examined the changes in the *E. coli* metabolome under D_2_O-induced stress. Therefore, the present study aimed to investigate the global metabolic response of multiple *E. coli* strains, both tolerant and susceptible phenotypes, to varying concentrations of D_2_O, providing new insights into the systems-level impact of isotopic stress on bacterial metabolism. LC-MS analysis identified 44 MSI Level 2 annotated metabolites showing an FDR-significant effect in the two-way ANOVA. Their abundance patterns across D_2_O concentrations and phenotype groups are presented in Fig. 6. Figure 5 presents an overview of the metabolic pathways affected by D_2_O exposure, mapped onto a comprehensive *E. coli* metabolic network for both tolerant and susceptible groups. To enable detailed examination, each affected pathway is presented individually, along with corresponding supplementary information that provides further context and interpretation (Fig. 6, S2). In the following exploration of biological function changes related to D_2_O exposure all metabolites directly mentioned are identified at MSI level 2. In cases where the uncertainty of the identification is higher, the MSI level is explicitly mentioned at each metabolite’s reference.

##### Central carbon metabolism

**Glucose utilisation and sugar-phosphate metabolism**

Glycolysis is a metabolic pathway that converts glucose into pyruvate, a process that occurs in the cytoplasm of all cells (Melkonian, & Schury, 2019).

In central carbon metabolism, the pooled susceptible group showed higher intracellular glucose abundance than the tolerant group at 0% D_2_O. At 40% D_2_O, mean glucose abundance decreased in susceptible isolates (fold change [FC] = 0.51), whereas the pooled tolerant mean showed little change. At 80% D_2_O, glucose increased relative to 40% D_2_O in the susceptible group, while the pooled tolerant mean remained relatively stable. However, significant differences were detected among tolerant strains at 80% D_2_O, indicating that the pooled tolerant value did not represent a uniform pattern across all three strains. Previous work has reported increased glucose uptake at high D_2_O concentrations, although glucose uptake was not directly measured here. (Opitz et al., 2019). Glucose-1-phosphate (G1P) is involved in sugar-phosphate metabolism, including glycogen- and UDP-glucose-associated processes, and can be reversibly interconverted with glucose-6-phosphate by phosphoglucomutase. The pooled susceptible group showed lower G1P abundance at 0% D_2_O; however, significant baseline variation was detected among the susceptible strains. The pooled increase observed at 40% D_2_O (FC = 1.21) should therefore be interpreted as an average group-level pattern rather than a uniform response across all susceptible strains. At 80% D_2_O, mean G1P abundance decreased in both phenotype groups. These patterns may indicate perturbation of sugar-phosphate metabolism, but they do not directly demonstrate altered phosphoglucomutase activity or pathway flux. However, steady-state G1P abundance alone cannot determine whether these changes specifically reflect glycogen metabolism, cell-envelope-associated carbohydrate metabolism, or altered interconversion with glucose-6-phosphate.

At 0% D_2_O, the pooled PEP abundances were similar between the two phenotype groups. At 40% D_2_O, the pooled susceptible group showed a marked decrease in PEP abundance (FC = 0.14). Significant strain effects were detected among tolerant isolates at 40% D_2_O and among both phenotype groups at 80% D_2_O. PEP patterns under these conditions were therefore interpreted as strain-dependent pooled averages rather than uniform phenotype-level responses. The reduced mean abundance in the susceptible group at 40% D_2_O may be consistent with perturbation of central-carbon metabolism, although its metabolic cause cannot be established from steady-state abundance measurements alone.

**TCA cycle-associated metabolites**

Several detected metabolites, including succinate and malate, are intermediates of the TCA cycle, whereas glutamate is metabolically connected to central carbon metabolism through *α*-ketoglutarate but also participates in multiple other cellular processes. Several core TCA intermediates, including oxaloacetate, isocitrate, *α*-ketoglutarate, succinyl-CoA and fumarate, were not detected. Therefore, the observed metabolite abundances are interpreted as TCA cycle-associated patterns rather than direct measures of TCA cycle activity or flux. Glutamate showed phenotype-associated patterns in the pooled data. At 40% D_2_O, mean glutamate abundance decreased in the tolerant group but showed little change in the susceptible group. However, significant baseline variation was detected among susceptible strains at 0% D_2_O, indicating that the pooled comparison between 0% and 40% D_2_O may not represent all three susceptible strains equally. At 80% D_2_O, mean glutamate abundance decreased in the susceptible group, whereas the tolerant group remained relatively stable (Figs. 6 and S2). These values are therefore interpreted as average group-level patterns rather than uniform responses across all strains. As glutamate participates in multiple cellular processes, including amino acid and nitrogen metabolism and stress-associated responses, its abundance changes cannot be attributed specifically to TCA cycle activity. Potential links between these patterns, glutamate dehydrogenase (GDH) and D_2_O-associated kinetic isotope effects are discussed in Supplementary Information. Succinate showed contrasting pooled abundance patterns between the phenotype groups. At 40% D_2_O, mean succinate abundance increased in susceptible isolates but decreased in tolerant isolates (Figs. 5 and S2). However, significant strain effects were detected within both phenotype groups at 0% D_2_O, within tolerant isolates at 40% D_2_O, and within both groups at 80% D_2_O. The pooled succinate values should therefore be interpreted as average patterns rather than uniform responses across all isolates. FMN showed a larger mean decrease in tolerant isolates at 40% D_2_O, followed by increasing mean abundance at higher D_2_O concentrations. FMN is a flavin cofactor involved in multiple cellular reactions and is not specific to the TCA cycle. Its abundance is therefore interpreted more broadly as a D_2_O-associated change in flavin cofactor metabolism rather than as an indicator of succinate dehydrogenase activity, respiratory activity or TCA cycle flux. Mean malate abundance increased with D_2_O in the pooled phenotype groups and was higher in the pooled susceptible group at 40% and 80% D_2_O. However, significant differences among strains were detected within both phenotypes at 40% and 80% D_2_O. The pooled malate pattern therefore represents an average response and should not be generalised to every strain within either phenotype. Perturbation of malate-associated metabolism or malate dehydrogenase activity is one possible explanation, but this remains hypothetical because enzyme activity and metabolic flux were not measured. Taken together, these metabolite patterns suggest that tolerant isolates maintained comparatively more stable NAD-related cofactor and TCA-associated profiles under D_2_O exposure, whereas susceptible isolates showed earlier changes in succinate-, malate- and cofactor-associated metabolites and broader metabolic perturbation under high isotopic stress.

Taken together, the pooled central carbon profiles suggested phenotype-associated differences under D_2_O exposure. However, the strain-level sensitivity analysis identified significant heterogeneity in several central carbon metabolites, particularly succinate, malate and PEP. These results are therefore interpreted as average phenotype-level patterns rather than uniform metabolic responses across all constituent strains. The proposed effects on TCA-associated metabolism and redox-coupled reactions should consequently be regarded as hypotheses requiring direct flux and enzyme activity measurements. Expanded mechanistic interpretation and full fold-change values for all metabolites are provided in the Supplementary Information Section. 1.

##### Amino acid- and dipeptide-associated metabolism

We next examined D_2_O-associated changes in amino acids and putatively annotated dipeptide-related features (Figs. 5 and S2). These metabolites may participate in multiple processes, including amino acid metabolism, peptide metabolism and central carbon-associated reactions, but their abundance changes were not interpreted as direct indicators of TCA cycle activity or protein turnover. Valylphenylalanine and alanyltyrosine showed clear group-specific differences. At 40% D_2_O, the pooled susceptible group displayed sharp decreases in the putatively annotated valylphenylalanine and alanyltyrosine features (FC = 0.10 and FC = 0.08, respectively), accompanied by an increase in free valine. The change in valine may be relevant to branched-chain amino acid and transaminase-associated metabolism, given the role of BCAAs in energy metabolism and the contribution of E. coli aminotransferases, including AvtA, to the regulation of intracellular amino-acid pools (Pena-Soler et al., 2014; Reifenberg & Zimmer, 2024). These changes coincided with elevated succinate, whereas the tolerant group showed increased abundances of both dipeptide features. However, their origin remains uncertain, and the present steady-state data cannot distinguish between protein degradation, peptide uptake, synthesis or other processes. Although (Opitz et al., 2019) reported deuteration-associated changes in translational proteins, this does not establish that the increased valine observed here arose from protein degradation. As these dipeptides were identified at MSI Level 2 without authentic standards, they are interpreted cautiously as D_2_O-associated dipeptide-related features. At 80% D_2_O, both dipeptide features decreased in the tolerant group, whereas the susceptible group showed partial recovery compared with 40% D_2_O, although levels remained below baseline. These non-monotonic responses may reflect differences in amino acid- and peptide-associated metabolism under increasing D_2_O stress. However, they do not provide direct evidence of earlier peptide mobilisation in susceptible isolates or delayed peptide breakdown in tolerant isolates. Prolylleucine showed a similar pattern. Both groups exhibited comparable levels at baseline, but only susceptible isolates showed a marked decrease at 40% D_2_O (FC = 0.20), consistent with increased catabolism of proline- and leucine-containing peptides. At 80% D_2_O, prolylleucine levels increased in susceptible isolates relative to 40%, but remained below baseline, mirroring the intermediate accumulation observed for other dipeptides.

We also observed shifts in branched-chain amino acids (BCAAs). Threonine, a precursor of isoleucine (Cotton et al., 2020), was consistently higher in susceptible isolates across all D_2_O concentrations, and both groups showed a decreasing trend with increasing D_2_O. Isoleucine increased sharply in susceptible isolates at 40% D_2_O (FC = 1.51) and decreased in tolerant isolates, likely reflecting impaired downstream catabolism in susceptible strains due to reduced cofactor availability and ETC activity (Zhou et al., 2024). This interpretation is supported by (Opitz et al., 2019), who used proteomics to observe subtle downregulation of genes involved in isoleucine biosynthesis and degradation under D_2_O exposure.

Overall, the results demonstrate phenotype-associated differences in several amino acid- and putative dipeptide-related features under D_2_O exposure. However, the present steady-state metabolomics data do not establish whether these changes reflect protein degradation, peptide utilisation, altered uptake or pathway flux. Extended mechanistic interpretation of these patterns is provided in Supplementary Information Section. 2.

##### Cofactor, amino-acid and glutathione-associated metabolism under D_2_O exposure

Following the analysis of central-carbon-associated metabolites, we examined changes in metabolites associated with nicotinate and nicotinamide metabolism, arginine and proline metabolism, and glutathione and polyamine metabolism (Figs. 6 and S3). These pathway associations provide a framework for organising the observed metabolite changes but do not directly measure pathway flux, respiratory activity or cellular redox state.

**Nicotinate and nicotinamide metabolism**

In *E. coli*, nicotinate and nicotinamide are key precursors for NAD^+^ biosynthesis (Mehl et al., 2000). All detected metabolites in this pathway, nicotinate, nicotinamide, NAD^+^, and ADP-ribose, showed broadly similar responses to D_2_O (Figs. 5 and S3). Across all D_2_O concentrations, tolerant isolates exhibited higher relative abundances of nicotinate, nicotinamide, NAD⁺ and ADP-ribose than susceptible isolates. Importantly, these differences were already present at 0% D_2_O, indicating that they may represent baseline phenotype- or strain-associated differences rather than responses specific to D_2_O exposure. With increasing D_2_O, the relative abundances of these metabolites generally declined in both groups, indicating perturbation of NAD-related cofactor metabolism. Because NADH was not measured, the NAD⁺/NADH ratio and cellular redox state could not be determined from these data. Nevertheless, the coordinated decreases in NAD⁺, nicotinamide and nicotinate are consistent with the possibility that kinetic isotope effects perturb proton-coupled reactions within NAD⁺ metabolism, potentially including reactions associated with PncA. This interpretation remains hypothetical because enzyme activity and metabolic flux were not directly measured (Luo et al., 2023).

##### Arginine and proline metabolism

Within the arginine and proline pathway, proline can be produced from glutamate and reconverted to glutamate, thereby linking proline-associated metabolism with central carbon metabolism (Zhang et al., 2015). At 0% D_2_O, the pooled proline abundances were similar between the two phenotype groups; however, significant baseline variation was detected among susceptible strains. At 40% D_2_O, the pooled tolerant mean decreased, whereas the pooled susceptible mean increased. Because the susceptible baseline was heterogeneous, this increase is interpreted as an average group-level pattern rather than a uniform response across all susceptible strains. At 80% D_2_O, the pooled susceptible mean returned towards baseline, while the tolerant mean remained relatively stable. These changes may be associated with altered proline- or peptide-related metabolism, but they do not directly demonstrate increased reliance on peptide-derived carbon. By contrast, arginine showed an inverse trend. It was initially more abundant in susceptible isolates at 0% D_2_O, but at 40% D_2_O it decreased in susceptible isolates and increased in tolerant ones (Figs. 5 and S3). Arginine can feed into fumarate production and thereby contribute indirectly to TCA intermediates (Schneider et al., 1998), can be converted to glutamate via the urea cycle (Charlier, & Bervoets, 2019), and also serves as a precursor for putrescine via ornithine (Lillie et al., 2024). At 80% D_2_O both phenotypes showed higher arginine levels compared to 40% D_2_O, likely reflecting reduced utilisation under severe stress. This interpretation is supported by Opitz who reported that D_2_O limits arginine uptake (Opitz et al., 2019), suggesting that increased intracellular arginine at high D_2_O mainly reflects reduced consumption rather than increased synthesis.

##### Glutathione metabolism

Glutathione metabolism encompasses the synthesis, degradation, and utilisation of glutathione and related polyamines, which play key roles in detoxification and oxidative stress defence (Chen et al., 2024; Suzuki et al., 1999). Putrescine and spermidine link arginine and proline metabolism with glutathione-related stress responses. At 0% D_2_O, susceptible isolates produced less putrescine than tolerant isolates, but at 40% D_2_O this pattern reversed: susceptible isolates showed strong upregulation of putrescine, while tolerant isolates remained near baseline (Fig. 6 and S3). At 80% D_2_O, putrescine increased slightly in tolerant isolates and decreased from the 40% peak in susceptible isolates, although remaining above baseline, consistent with ongoing but metabolically constrained stress responses (Newo et al., 2004; Wang et al., 2024).

Spermidine did not show significant D_2_O-dependent changes (data not shown), in contrast to Opitz et al., (Opitz et al., 2019), who reported downregulation of spermidine metabolism genes under D_2_O exposure. Cadaverine, another stress-associated polyamine generated from lysine (Chen et al., 2024), showed even more pronounced differences. At 0% D_2_O, cadaverine was lower in susceptible isolates, but at 40% D_2_O, susceptible isolates displayed a dramatic increase (FC ≈ 101), whereas tolerant isolates showed a marked decrease. At 80% D_2_O, cadaverine increased in tolerant isolates, however at 80% in susceptible isolates, it declined compared the 40% peak, paralleling trends in other metabolites and suggesting a shift from robust stress induction to metabolically limited maintenance. These patterns are consistent with work showing that D_2_O can impair polyamine export and putrescine degradation, thereby promoting intracellular polyamine accumulation (Wang et al., 2024).

Lysine and methionine further supported enhanced protein turnover and stress adaptation in the three susceptible isolates. Lysine levels increased more strongly in susceptible compared to tolerant isolates at 40% D_2_O and remained elevated at 80% D_2_O, consistent with increased reliance on protein degradation to sustain cadaverine production, in line with reduced amino acid uptake under D_2_O reported by (Opitz et al., 2019). Methionine, a precursor of *S*-adenosylmethionine (SAM) and glycine involved in methylation and antioxidant responses (Ouyang et al., 2020), showed minor changes in tolerant isolates but a clearer decrease at 80% D_2_O in susceptible isolates, consistent with impaired ATP availability and reduced global protein synthesis under D_2_O stress (Opitz et al., 2019; Wang et al., 2024).

Glutathione-related metabolites showed different abundance patterns between the pooled phenotype groups. The tolerant group showed higher baseline glutathione abundance, whereas at 40% D_2_O, the susceptible group showed a marked increase and the tolerant group showed a decrease. At 80% D_2_O, glutathione abundance decreased further in the tolerant group and declined from its 40% D_2_O maximum in the susceptible group, although it remained above the susceptible baseline. Changes in γ-glutamyl–amino acids, prolylglycine, L-γ-glutamyl-L-amino acid and 5-oxoproline were broadly consistent with perturbation of glutathione- and γ-glutamyl-cycle-associated metabolism under D_2_O. However, 5-oxoproline showed significant variation among susceptible strains at 80% D_2_O, indicating that its pooled abundance did not represent a uniform susceptible-group response under this condition. These patterns may reflect altered antioxidant-associated metabolism; however, steady-state metabolite abundances do not directly measure glutathione turnover or pathway flux. Furthermore, oxidative stress and cellular redox state were not directly determined because oxidised glutathione, the GSH/GSSG ratio and direct oxidative-stress markers were not measured. Taken together, these data show that susceptible isolates exhibit more pronounced changes in polyamine, glutathione and redox-associated metabolites under D_2_O, whereas tolerant isolates generally show more moderate changes. These patterns are consistent with differential antioxidant- and stress-associated metabolism, although they do not directly establish cellular redox state or oxidative stress. Detailed mechanistic interpretations of these pathway-level responses are provided in Supplementary Information Section. 3.

##### Nucleotides and cofactor metabolisms

**Purine metabolism**

Purine metabolism involves the synthesis, recycling, and breakdown of purine bases, which are essential for nucleic acids and cellular energy balance (Xi et al., 2000). Starting from inosine monophosphate (IMP), both tolerant and susceptible isolates showed similar baseline levels at 0% D_2_O (Fig. 6). With increasing D_2_O, IMP and its downstream nucleotides xanthosine 5′-monophosphate (XMP), guanosine monophosphate (GMP), and adenosine monophosphate (AMP) all showed progressive decreases in both groups (Figs. 5 and S4), consistent with elevated purine turnover under stress. Susceptible isolates generally exhibited earlier and more pronounced reductions (e.g. IMP FC = 0.80 at 40% D_2_O *vs*. 1.02 in tolerant isolates), suggesting faster mobilisation of purine intermediates, whereas tolerant isolates showed a more delayed and moderate response.

Although adenosine diphosphate (ADP) did not reach statistical significance, its behaviour followed similar trends (data not shown), supporting the interpretation of D_2_O-imposed energy stress. In contrast, adenosine itself showed a distinct pattern. At 0% D_2_O, susceptible isolates had much lower adenosine compared to tolerant isolates (Figs. 5 and S4). At 40% D_2_O, adenosine decreased in tolerant isolates (FC = 0.56) but increased markedly in susceptible isolates (FC = 3.03), suggesting differential handling of this metabolite. One explanation is that susceptible strains accumulate adenosine due to increased AMP breakdown and reduced conversion to inosine, potentially reflecting impaired activity or regulation of adenosine deaminase (Ada). While strain-specific differences in Add expression in *E. coli* have not been reported, tolerant strains may maintain more effective Add function under D_2_O exposure.

Hypoxanthine, a downstream product of inosine, broadly mirrored the behaviour of IMP, AMP, XMP, and GMP (Figs. 5 and S4), indicating enhanced flux through the purine salvage pathway. Together, these findings suggest that both isolate types, particularly the susceptible strains, mobilise purine pools to support ATP and GTP synthesis and thereby help sustain TCA cycle activity under D_2_O stress (Mrnjavac, & Martin, 2025). Tolerant strains appear to follow a similar strategy but with a delayed and more controlled response, consistent with their more stable TCA output and reduced reliance on nucleotide catabolism as an energy buffer, in line with patterns seen in dipeptide metabolism. These conclusions are supported by Opitz who reported upregulation of genes involved in deoxyribonucleotide catabolism in D_2_O-exposed *E. coli* (Opitz et al., 2019), suggesting that purine degradation and salvage form part of a broader compensatory mechanism to sustain energy production. A secondary, less likely explanation is increased nucleotide demand for DNA and RNA synthesis during mid-exponential growth, but the consistent D_2_O-dependent declines across purine intermediates favour an energy-focused interpretation.

**Pyrimidine metabolism**

Pyrimidine metabolism encompasses the biosynthesis, salvage, degradation, and transport of pyrimidine nucleotides and nucleosides (Figure S4), which are crucial for DNA and RNA synthesis (Garavito et al., 2015). Orotate, a key precursor in *de novo* UMP synthesis, was considerably higher in tolerant isolates at 0% D_2_O (Figs. 5 and S4), indicating more active or stable pyrimidine biosynthesis in this group. At 40% D_2_O, both phenotypes showed a similar decrease in orotate (FC = 0.43), consistent with reduced production and reflecting the high ATP demand of carbamoyl phosphate synthesis, a precursor to orotate (Charlier et al., 2018). The dependence of this pathway on aspartate derived from OAA further links orotate levels to central metabolism.

At 80% D_2_O, orotate decreased further in tolerant isolates (FC = 0.67), whereas susceptible isolates showed a relative increase from 40% D_2_O (FC = 1.32), suggesting a bottleneck in the conversion of orotate to UMP. This step requires 5-phosphoribosyl-1-pyrophosphate (PRPP) (Huang, & Graves, 2003), which may become limiting under D_2_O-induced metabolic stress. In the pooled data, UMP abundance was higher in tolerant isolates under control conditions, decreased in both phenotype groups at 40% D_2_O, and showed relatively little further mean change at 80% D_2_O. However, significant strain variation was detected among tolerant isolates at 40% D_2_O and within both phenotype groups at 80% D_2_O. The later-condition UMP patterns should therefore be interpreted as strain-dependent pooled averages rather than uniform phenotype responses. Pathway saturation or regulatory restriction of de novo pyrimidine synthesis represents one possible explanation, but this cannot be established from the current steady-state data. Uracil, a degradation product of UMP and a marker of RNA turnover (Berens et al., 1995), was consistently higher in tolerant isolates (Figs. 5and S4), supporting a more dynamic nucleotide metabolism at baseline. At 40% D_2_O, uracil decreased in both phenotypes (FC = 0.66 in tolerant; 0.52 in susceptible isolates), suggesting reduced RNA turnover or impaired salvage. At 80% D_2_O, uracil declined modestly in tolerant isolates but increased in susceptible ones (FC = 1.78), consistent with enhanced RNA degradation under stress. This interpretation is supported by Opitz et al. who reported upregulation of RNA catabolic processes, ribonuclease activity, and mRNA decay in D_2_O-treated *E. coli* (Opitz et al., 2019).

Cytidine monophosphate (CMP) displayed complementary behaviour (Figure S4). CMP was lower in tolerant strains across conditions, suggesting more efficient utilisation for CTP synthesis or routing into degradation and salvage, preventing its accumulation. At 40% D_2_O, CMP decreased in tolerant isolates (FC = 0.68) but remained unchanged in susceptible isolates (FC = 0.94), consistent with earlier activation of salvage or reduced RNA synthesis in the tolerant group. At 80% D_2_O, both phenotypes showed lower CMP, with susceptible isolates displaying a delayed decline to similar levels (FC = 0.68), suggesting late engagement of salvage pathways or collapse of RNA synthesis under severe stress. Given the ATP-intensive nature of CTP synthesis, shunting CMP toward degradation (CMP → cytidine → uridine → uracil) represents a more energetically favourable route under D_2_O-imposed energy limitation.

Overall, purine and pyrimidine pathways show broadly similar trends of progressive nucleotide depletion with D_2_O, particularly in susceptible isolates, consistent with their use as flexible energy and biosynthetic buffers under isotopic stress. Detailed mechanistic interpretations of these pathway-level responses are provided in supplementary information Section. 4.

## Conclusion

This study provides a systems-level characterisation of intracellular metabolite-abundance changes in *E. coli* following D_2_O exposure using untargeted LC-MS metabolomics and pathway analysis. Forty-four MSI Level 2 annotated metabolites showed significant D_2_O-associated changes and were represented across multiple overlapping metabolic categories, including central carbon-associated, amino acid, nucleotide, nicotinate and nicotinamide, glutathione and polyamine metabolism. Because these data represent steady-state metabolite abundances, they do not directly establish altered metabolic flux, enzyme inhibition, respiratory activity, oxidative stress or cellular redox state.

Across the pooled phenotype groups, susceptible and tolerant isolates showed different abundance patterns for several D_2_O-responsive metabolites, while the strain-level sensitivity analysis identified significant within-phenotype heterogeneity for a subset of metabolites. The findings therefore support D_2_O-associated metabolite perturbations and broader phenotype-associated patterns rather than uniform mechanistic responses across all strains. Potential contributions from altered central carbon, cofactor, nucleotide, amino acid and glutathione-associated metabolism provide hypotheses for D_2_O tolerance that require targeted flux, enzyme activity, respiratory and redox measurements for validation.

While untargeted metabolomics provided broad pathway-level coverage of D_2_O-induced perturbations, additional layers of information are needed to resolve adaptation mechanisms fully. A further limitation is that intracellular metabolite abundances were normalised using OD₆₀₀ alone, which may not fully reflect condition-dependent differences in cell number or biomass under D_2_O exposure. Therefore, the findings should be interpreted as relative OD₆₀₀-normalised abundances. Future studies should incorporate time-resolved sampling with ^13^C isotope labelling of metabolites to capture the dynamics of early and late responses, and include extracellular metabolomics to assess uptake, secretion, and medium-driven metabolic interactions. Expanding chemical coverage with complementary platforms such as GC-MS and validating metabolites using authentic standards would further enhance annotation confidence. Integrating metabolomics with transcriptomics, proteomics, lipidomics, and genome sequencing will be crucial for linking metabolic shifts to underlying regulatory and genetic determinants. Enzyme activity assays will also be important to confirm the functional impact of D_2_O on hydrogen-transfer reactions, redox cofactors, and membrane-associated processes.

Together, this study established a foundational metabolic framework for understanding how D_2_O perturbs bacterial physiology and identifies pathways that underlie isotopic stress resilience. These insights provide a basis for optimising stable isotope probing, improving interpretation of D_2_O-based analytical tools, and engineering microbial systems with enhanced robustness for biotechnology and synthetic biology applications.

## Supplementary Information

Below is the link to the electronic supplementary material.

Supplementary material 1 (DOCX 1400.5 kb)

## Data Availability

The metabolomics data generated in this study have been deposited in the MetaboLights repository under the study identifier MTBLS14330.
