# Supplementary material for: Metabolic profiling of Escherichia coli under deuterium oxide-induced stress
Source: Metabolomics. 2026 Sep 23;22(5):156. doi: 10.1007/s11306-026-02532-3 (PMC13601189; doi:10.1007/s11306-026-02532-3)
Supplement: Supplementary file 1 [file 11306_2026_2532_MOESM1_ESM.docx]

**Metabolic Profiling of *Escherichia coli* Under Deuterium Oxide-Induced Stress: A Metabolomics Approach**

Sahand Shams^1^, Ivayla Roberts^1^, Ahmed Nusaim Satheek Rehuman^1^, Nigel Gotts^1^, Yun Xu^1^, Shwan Ahmed^1,2^, Fabian Jourdan^3^, Catherine Winder^1^, Adam Burke^1^, Royston Goodacre^1^, Howbeer Muhamadali^1*^

^1^ Centre of Metabolomics Research, Department of Biochemistry, Cell and Systems Biology, Institute of Systems, Molecular and Integrative Biology, University of Liverpool, Liverpool, United Kingdom.

^2^ Department of Environment and Quality Control, Kurdistan Institution for Strategic Studies and Scientific Research, Kurdistan Region, Iraq.

^3^ MetaboHUB-Metatoul, National Infrastructure of Metabolomics and Fluxomics, Toulouse, France

# Supplementary Information

**Table S1: Composition of M9 minimal medium used for the growth of *E. coli* K-12 MG1655**

| Chemical Compounds | Chemical Formula | Concentration (gL^-1^) |
| --- | --- | --- |
| Ammonium chloride | NH_4_Cl | 1 |
| Magnesium sulfate heptahydrate | MgSO_4_.7H_2_O | 0.5 |
| Sodium hydrogen phosphate | Na_2_HPO_4_ | 3 |
| Potassium dihydrogen phosphate | KH_2_PO_4_ | 3 |
| Ferrous sulfate heptahydrate | FeSO_4_.7H_2_O | 0.001 |
| Calcium chloride | CaCl_2_ | 0.001 |
|  |  |  |

Here, nicotinic acid is presented as a representative example of the isotope-incorporation output generated by Compound Discoverer (CD). The overlaid chromatographic profiles showed comparable retention times across the 0%, 40% and 80% D₂O groups, suggesting that detected deuterium incorporation did not produce a clear chromatographic shift for this metabolite (Figure S1-A). The MS1 spectrum of the pooled sample showed a more complex pattern than a conventional monoisotopic M, M+1 and M+2 isotope envelope. For example, the signal at *m/z* 125.04266 was assigned as the natural-abundance M+1 isotopic peak of nicotinic acid, primarily corresponding to one ^13^C substitution, whereas the nearby signal at *m/z* 125.04545 was assigned as the M+1 isotopologue containing one incorporated deuterium atom (Figure S1-B). CD grouped these related signals according to the parameters defined in the Analyse Labelled Compounds node. The resulting isotopologue distribution is shown in Figure S1-C. Samples grown in 0% D₂O were dominated by the M+0 form, whereas samples grown in 40% and 80% D₂O showed increasing relative contributions from higher M+n isotopologues. Blue, orange and cyan represent the 0%, 40% and 80% D₂O groups, respectively.

**Figure S1.** Representative Compound Discoverer output for deuterium-isotopologue analysis of nicotinic acid. (A) Overlaid chromatographic peaks across 0%, 40% and 80% D₂O conditions, showing no clear retention-time shift. (B) Representative pooled-sample MS1 spectrum showing the natural-abundance M+1 peak and the nearby M+1 deuterium-labelled isotopologue. (C) Relative distribution of nicotinic acid isotopologues from M+0 to M+5. Blue, orange and cyan represent 0%, 40% and 80% D₂O, respectively.

**Table S2.** List of 45 metabolites that were affected significantly in at least one of the by D_2_O concentrations between susceptible and less-susceptible isolates (P-value<0.05).

|  | Metabolites | Polarity | *m/z* | FDR | Database Identifier |
| --- | --- | --- | --- | --- | --- |
| 1 | **Putrescine** | **Positive** | **89.10727** | **4.63E-05** | **KEGG:** [**C00134**](https://www.kegg.jp/dbget-bin/www_bget?cpd:C00134) |
| 2 | **Cadaverine** | **Positive** | **103.12295** | **2.50E-03** | **KEGG:** [**C01672**](https://www.kegg.jp/dbget-bin/www_bget?cpd:C01672) |
| 3 | **Pipecolic acid** | **Positive** | **130.08628** | **3.33E-02** | **KEGG:** [**C00408**](https://www.kegg.jp/dbget-bin/www_bget?cpd:C00408) |
| 4 | **Arginine** | **Positive** | **175.11892** | **4.85E-02** | **KEGG:** [**C00062**](https://www.kegg.jp/dbget-bin/www_bget?cpd:C00062) |
| 5 | **Threonine** | **Positive** | **120.06548** | **2.60E-09** | **KEGG:** [**C00188**](https://www.kegg.jp/dbget-bin/www_bget?cpd:C00188) |
| 6 | **Glutamic acid** | **Positive** | **148.06033** | **4.89E-03** | **KEGG:** [**C00025**](https://www.kegg.jp/dbget-bin/www_bget?cpd:C00025) |
| 7 | **Glucose 1-phosphate** | **Positive** | **261.037** | **3.56E-04** | **KEGG:** [**C00103**](https://www.kegg.jp/dbget-bin/www_bget?cpd:C00103) |
| 8 | ***N*-Acetylneuraminic acid** | **Positive** | **310.1131** | **4.76E-04** | **KEGG:** [**C00270**](https://www.kegg.jp/dbget-bin/www_bget?cpd:C00270) |
| 9 | **Proline** | **Positive** | **116.0705** | **2.72E-02** | **KEGG:** [**C00148**](https://www.kegg.jp/dbget-bin/www_bget?cpd:C00148) |
| 10 | **Cytidine monophosphate** | **Positive** | **324.05897** | **3.43E-03** | **KEGG:** [**C00055**](https://www.kegg.jp/dbget-bin/www_bget?cpd:C00055) |
| 11 | ***Υ*-Glutamyl-glutamic acid** | **Positive** | **277.10305** | **7.21E-15** | **KEGG:** [**C05282**](https://www.genome.jp/dbget-bin/www_bget?cpd:C05282) |
| 12 | **Prolylglycine** | **Positive** | **173.0921** | **2.78E-05** | [**HMDB:**](https://www.kegg.jp/dbget-bin/www_bget?cpd:C02612) [**HMDB0000721**](https://www.hmdb.ca/metabolites/HMDB0000721) |
| 13 | **Orotic acid** | **Positive** | **157.02439** | **0.047983** | **KEGG:** [**C00295**](https://www.kegg.jp/dbget-bin/www_bget?cpd:C00295) |
| 14 | **Valine** | **Positive** | **118.08615** | **2.96E-02** | **KEGG:** [**C00183**](https://www.kegg.jp/dbget-bin/www_bget?cpd:C00183) |
| 15 | **Acetylarginine** | **Positive** | **217.12952** | **8.95E-03** | **HMDB:** [**HMDB0004620**](https://www.hmdb.ca/metabolites/HMDB0004620) |
| 16 | **Uracil** | **Positive** | **113.03452** | **1.83E-02** | **KEGG:** [**C00106**](https://www.kegg.jp/dbget-bin/www_bget?cpd:C00106) |
| 17 | **Guanosine monophosphate** | **Positive** | **364.06513** | **1.19E-02** | **KEGG:** [**C00144**](https://www.kegg.jp/dbget-bin/www_bget?cpd:C00144) |
| 18 | **Inosine monophosphate** | **Positive** | **349.05428** | **3.99E-02** | **KEGG:** [**C00130**](https://www.kegg.jp/dbget-bin/www_bget?cpd:C00130) |
| 19 | **Hypoxanthine** | **Positive** | **137.04579** | **1.71E-02** | **KEGG:** [**C00262**](https://www.kegg.jp/dbget-bin/www_bget?cpd:C00262) |
| 20 | **Glutathione** | **Positive** | **308.09094** | **3.70E-02** | **KEGG:** [**C00051**](https://www.kegg.jp/dbget-bin/www_bget?cpd:C00051) |
| 21 | **Nicotinic acid** | **Positive** | **124.03929** | **8.49E-04** | **KEGG:** [**C00253**](https://www.kegg.jp/dbget-bin/www_bget?cpd:C00253) |
| 22 | **Xanthosine monophosphate** | **Positive** | **365.04897** | **5.55E-04** | **KEGG:** [**C00655**](https://www.kegg.jp/dbget-bin/www_bget?cpd:C00655) |
| 23 | **Methionine** | **Positive** | **150.05827** | **1.93E-02** | **KEGG:** [**C00073**](https://www.kegg.jp/dbget-bin/www_bget?cpd:C00073) |
| 24 | **Nicotinamide adenine dinucleotide** | **Positive** | **664.11599** | **1.04E-02** | **KEGG:** [**C00003**](https://www.kegg.jp/dbget-bin/www_bget?cpd:C00003) |
| 25 | **ADP-ribose** | **Positive** | **542.06885** | **1.26E-05** | **KEGG:** [**C00301**](https://www.kegg.jp/dbget-bin/www_bget?cpd:C00301) |
| 26 | **Nicotinamide** | **Positive** | **123.05525** | **1.29E-02** | **KEGG:** [**C00153**](https://www.kegg.jp/dbget-bin/www_bget?cpd:C00153) |
| 27 | **Adenosine monophosphate** | **Positive** | **332.07534** | **7.52E-03** | **KEGG:** [**C00020**](https://www.genome.jp/dbget-bin/www_bget?cpd:C00020) |
| 28 | **Tyrosine** | **Positive** | **182.08113** | **3.83E-02** | **KEGG:** [**C00082**](https://www.kegg.jp/dbget-bin/www_bget?cpd:C00082) |
| 29 | **Isoleucine** | **Positive** | **132.10182** | **1.33E-02** | **KEGG:** [**C00407**](https://www.kegg.jp/dbget-bin/www_bget?cpd:C00407) |
| 30 | **Adenosine** | **Positive** | **268.10405** | **3.31E-02** | **KEGG:** [**C00212**](https://www.kegg.jp/dbget-bin/www_bget?cpd:C00212) |
| 31 | **Alanyltyrosine** | **Positive** | **253.1183** | **2.06E-04** | [**HMDB:**](https://www.kegg.jp/dbget-bin/www_bget?cpd:C03508) [**HMDB0304776**](https://hmdb.ca/metabolites/HMDB0304776) |
| 32 | **Phenylalanine** | **Positive** | **166.08615** | **1.92E-02** | **KEGG:** [**C00079**](https://www.kegg.jp/dbget-bin/www_bget?cpd:C00079) |
| 33 | **Prolylleucine** | **Positive** | **229.15456** | **6.11E-06** | **HMDB:** [**HMDB0253028**](https://hmdb.ca/metabolites/HMDB0253028) |
| 34 | **Valylphenylalanine** | **Positive** | **265.15473** | **3.55E-03** | **HMDB:** [**HMDB0029134**](https://hmdb.ca/metabolites/HMDB0029134) |
| 35 | **Cyclo(leucylprolyl)** | **Positive** | **211.14409** | **1.87E-02** | **HMDB:** [**HMDB0034276**](https://www.hmdb.ca/metabolites/HMDB0034276) |
| 36 | **Cyclo(phenylalanyl-prolyl)** | **Positive** | **245.12853** | **2.55E-13** | **HMDB:** [**HMDB0250631**](https://www.hmdb.ca/metabolites/HMDB0250631) |
| 37 | **Lysine** | **Positive** | **147.11278** | **3.62E-05** | **KEGG:** [**C00047**](https://www.kegg.jp/dbget-bin/www_bget?cpd:C00047) |
| 38 | **Glucose** | **Negative** | **179.05657** | **1.37E-04** | **KEGG:**[**C00031**](https://www.kegg.jp/dbget-bin/www_bget?cpd:C00031) |
| 39 | **Malic acid** | **Negative** | **133.01456** | **9.35E-03** | **KEGG:**[**C00149**](https://www.kegg.jp/dbget-bin/www_bget?cpd:C00149) |
| 40 | **Uridine monophosphate** | **Negative** | **323.02951** | **4.32E-02** | **KEGG:**[**C00105**](https://www.kegg.jp/dbget-bin/www_bget?cpd:C00105) |
| 41 | **Phosphoenolpyruvic acid** | **Negative** | **166.97552** | **9.90E-05** | **KEGG:**[**C00074**](https://www.kegg.jp/dbget-bin/www_bget?cpd:C00074) |
| 42 | **5-Oxoproline** | **Negative** | **128.03555** | **1.05E-14** | **KEGG:**[**C01879**](https://www.kegg.jp/dbget-bin/www_bget?cpd:C01879) |
| 43 | **Succinic acid** | **Negative** | **117.01946** | **4.81E-02** | **KEGG:**[**C00042**](https://www.kegg.jp/dbget-bin/www_bget?cpd:C00042) |
| 44 | **Flavin mononucleotide** | **Negative** | **455.09854** | **2.40E-02** | **KEGG:**[**C00061**](https://www.kegg.jp/dbget-bin/www_bget?cpd:C00061) |

**Table S3.** Network-based pathway enrichment analysis of *Escherichia coli* metabolism using MetExplore

| **Pathway** | **KEGG ID** | **Reactions (n)** | **Enzyme-associated (%)** | **Coverage (%)** | **Mapped Metabolites (n)** | ***p*-value** | **FDR (BH)** |
| --- | --- | --- | --- | --- | --- | --- | --- |
| **Biosynthesis of secondary metabolites** | eco01110 | 382 | 100 | 6.19 | 24 | 1.08169E-06 | 4.97577E-05 |
| **Aminoacyl-tRNA biosynthesis** | eco00970 | 23 | 100 | 15.49 | 11 | 6.65757E-07 | 4.97577E-05 |
| **Biosynthesis of amino acids** | eco01230 | 113 | 100 | 10.37 | 14 | 2.20234E-06 | 6.75383E-05 |
| **Biosynthesis of cofactors** | eco01240 | 178 | 100 | 7.44 | 18 | 5.98127E-06 | 0.000110055 |
| **Nucleotide metabolism** | eco01232 | 111 | 100 | 12.64 | 11 | 5.51003E-06 | 0.000110055 |
| **Purine metabolism** | eco00230 | 91 | 100 | 9.09 | 9 | 0.000657676 | 0.00732868 |
| **Methane metabolism** | eco00680 | 21 | 100 | 13.95 | 6 | 0.000631072 | 0.00732868 |
| **Alanine, aspartate and glutamate metabolism** | eco00250 | 27 | 100 | 13.64 | 6 | 0.000716936 | 0.00732868 |
| **Pantothenate and CoA biosynthesis** | eco00770 | 20 | 100 | 14.63 | 6 | 0.0004835 | 0.007413659 |
| **Valine, leucine and isoleucine biosynthesis** | eco00290 | 20 | 100 | 14.29 | 5 | 0.001699601 | 0.010424222 |
| **Arginine and proline metabolism** | eco00330 | 32 | 100 | 11.76 | 6 | 0.001600745 | 0.010424222 |
| **Nicotinate and nicotinamide metabolism** | eco00760 | 23 | 100 | 14.29 | 5 | 0.001699601 | 0.010424222 |
| **Metabolic pathways** | eco01100 | 1348 | 100 | 3.16 | 35 | 0.001382131 | 0.010596336 |
| **Lysine degradation** | eco00310 | 16 | 100 | 15.15 | 5 | 0.001290699 | 0.010596336 |
| **Novobiocin biosynthesis** | eco00401 | 2 | 100 | 33.33 | 3 | 0.001246149 | 0.010596336 |
| **Glutathione metabolism** | eco00480 | 20 | 100 | 13.16 | 5 | 0.002482283 | 0.014273127 |
| **Tyrosine metabolism** | eco00350 | 10 | 100 | 16.67 | 4 | 0.00290103 | 0.015699689 |
| **Pyruvate metabolism** | eco00620 | 41 | 100 | 10.34 | 6 | 0.003148008 | 0.016089817 |
| **Phenylalanine, tyrosine and tryptophan biosynthesis** | eco00400 | 25 | 100 | 11.63 | 5 | 0.004328951 | 0.018942332 |
| **Other carbon fixation pathways** | eco00720 | 21 | 100 | 11.63 | 5 | 0.004328951 | 0.018942332 |
| **beta-Alanine metabolism** | eco00410 | 8 | 100 | 14.81 | 4 | 0.004529688 | 0.018942332 |
| **Pyrimidine metabolism** | eco00240 | 71 | 100 | 8.64 | 7 | 0.003925844 | 0.019009348 |
| **Valine, leucine and isoleucine degradation** | eco00280 | 12 | 100 | 13.79 | 4 | 0.005905055 | 0.023620219 |
| **Glycolysis / Gluconeogenesis** | eco00010 | 32 | 100 | 10.42 | 5 | 0.007002711 | 0.025882364 |
| **Porphyrin metabolism** | eco00860 | 29 | 100 | 8.82 | 6 | 0.007033251 | 0.025882364 |
| **Lysine biosynthesis** | eco00300 | 14 | 100 | 12.9 | 4 | 0.007535818 | 0.026665201 |
| **Phenylalanine metabolism** | eco00360 | 31 | 100 | 9.43 | 5 | 0.010672819 | 0.036366644 |
| **Glyoxylate and dicarboxylate metabolism** | eco00630 | 33 | 100 | 9.26 | 5 | 0.011540658 | 0.036918508 |
| **Citrate cycle (TCA cycle)** | eco00020 | 19 | 100 | 11.43 | 4 | 0.011637356 | 0.036918508 |
| **Carbon metabolism** | eco01200 | 102 | 100 | 6.73 | 7 | 0.015454381 | 0.047393435 |

**Table S4. Statistical results and pathway/category memberships of the 44 significantly altered annotated (MSI 2) metabolites.**

| **Metabolite** | **Ion mode(s)** | **D₂O effect BH-FDR p-value** | **Phenotype effect BH-FDR *p*-value** | **Pathway/category membership(s)** |
| --- | --- | --- | --- | --- |
| 5-Oxoproline | ESI+ | 4.48E-12 (Sig.) | 6.49E-04 (Sig.) | Glutathione metabolism |
| Acetylarginine | ESI+ | 0.0089 (Sig.) | 0.1828 (NS) | Not assigned to selected pathway categories |
| Adenosine | ESI+ | 0.0331 (Sig.) | 7.89E-12 (Sig.) | Purine metabolism |
| ADP-ribose | ESI+ | 1.26E-05 (Sig.) | 5.90E-04 (Sig.) | Nicotinate/nicotinamide-associated metabolism |
| Alanyltyrosine | ESI+ | 2.06E-04 (Sig.) | 9.94E-04 (Sig.) | Dipeptide/amino-acid-associated metabolism |
| AMP | ESI+ | 0.0075 (Sig.) | 0.1488 (NS) | Purine metabolism |
| Arginine | ESI+ | 0.0193 (Sig.) | 0.0095 (Sig.) | Arginine and proline metabolism |
| Cadaverine | ESI+ | 0.0025 (Sig.) | 0.1593 (NS) | Glutathione metabolism |
| CMP | ESI+ | 0.0034 (Sig.) | 4.62E-17 (Sig.) | Pyrimidine metabolism |
| Cyclo(leucylprolyl) | ESI+ | 0.0187 (Sig.) | 0.2672 (NS) | Dipeptide/amino-acid-associated metabolism |
| Cyclo(phenylalanyl-prolyl) | ESI+ | 2.55E-13 (Sig.) | 0.0088 (Sig.) | Dipeptide/amino-acid-associated metabolism |
| Flavin mononucleotide | ESI− | 0.02400 (Sig.) | 0.0349 (Sig.) | Flavin/cofactor-associated metabolite, not assigned to selected pathway categories |
| Glucose | ESI− | 1.37E-04 (Sig.) | 0.0225 (Sig.) | Glucose/sugar-phosphate metabolism |
| Glucose 1-phosphate | ESI− | 0.0113 (Sig.) | 0.0668 (NS) | Glucose/sugar-phosphate metabolism |
| Glutamate | ESI+ | 0.0049 (Sig.) | 0.0014 (Sig.) | TCA cycle; Arginine and proline metabolism; Glutathione metabolism |
| GMP | ESI+ | 0.0119 (Sig.) | 0.1772 (NS) | Purine metabolism |
| Hypoxanthine | ESI+ | 0.0171 (Sig.) | 0.0033 (Sig.) | Purine metabolism |
| IMP | ESI+ | 0.0399 (Sig.) | 0.0014 (Sig.) | Purine metabolism |
| Isoleucine | ESI+ | 0.0133 (Sig.) | 0.0060 (Sig.) | Valine, leucine and isoleucine-associated metabolism |
| Lysine | ESI+ | 3.62E-05 (Sig.) | 0.0153 (Sig.) | Glutathione metabolism |
| Malate | ESI− | 0.0094 (Sig.) | 5.95E-05 (Sig.) | TCA cycle; Valine, leucine and isoleucine-associated metabolism |
| Methionine | ESI+ | 0.0193 (Sig.) | 0.0046 (Sig.) | Glutathione metabolism |
| N-Acetylneuraminic acid | ESI+ | 4.76E-04 (Sig.) | 1.69E-08 (Sig.) | Not assigned to selected pathway categories |
| NAD+ | ESI+ | 0.0104 (Sig.) | 4.09E-08 (Sig.) | TCA cycle; Nicotinate/nicotinamide-associated metabolism |
| Nicotinamide | ESI+ | 0.0129 (Sig.) | 1.60E-05 (Sig.) | Nicotinate/nicotinamide-associated metabolism |
| Nicotinic acid | ESI+ | 8.49E-04 (Sig.) | 2.32E-05 (Sig.) | Nicotinate/nicotinamide-associated metabolism |
| Orotate | ESI+ | 0.0480 (Sig.) | 4.64E-05 (Sig.) | Pyrimidine metabolism |
| Phenylalanine | ESI+ | 0.0192 (Sig.) | 0.0521 (NS) | Amino-acid-associated metabolite; not assigned to selected pathway categories |
| Phosphoenolpyruvate (PEP) | ESI− | 9.90E-05 (Sig.) | 0.1218 (NS) | Glucose/sugar-phosphate metabolism; Valine, leucine and isoleucine-associated metabolism |
| Pipecolic acid | ESI+ | 0.0333 (Sig.) | 0.0096 (Sig.) | Amino-acid-associated metabolite; not assigned to selected pathway categories |
| Proline | ESI+ | 0.0272 (Sig.) | 0.0033 (Sig.) | Arginine and proline metabolism |
| Prolylglycine | ESI+ | 2.78E-05 (Sig.) | 0.0061 (Sig.) | Dipeptide/amino-acid-associated metabolism; glutathione-associated metabolism |
| Prolylleucine | ESI+ | 1.55E-04 (Sig.) | 1.68E-04 (Sig.) | Dipeptide/amino-acid-associated metabolism |
| Putrescine | ESI+ | 4.63E-05 (Sig.) | 0.0022 (Sig.) | Arginine and proline metabolism; Glutathione metabolism |
| Reduced glutathione (GSH) | ESI+ | 0.0370 (Sig.) | 0.1827 (NS) | Glutathione metabolism |
| Succinate | ESI− | 0.0481 (Sig.) | 0.1295 (NS) | TCA cycle; Nicotinate/nicotinamide-associated metabolism; Valine, leucine and isoleucine-associated metabolism |
| Threonine | ESI+ | 2.60E-09 (Sig.) | 8.93E-05 (Sig.) | Valine, leucine and isoleucine-associated metabolism |
| Tyrosine | ESI+ | 1.59E-04 (Sig.) | 0.1990 (NS) | Amino-acid-associated metabolite; not assigned to selected pathway categories |
| UMP | ESI− | 0.0432 (Sig.) | 0.0050 (Sig.) | Pyrimidine metabolism |
| Uracil | ESI+ | 0.0183 (Sig.) | 8.74E-22 (Sig.) | Pyrimidine metabolism |
| Valine | ESI+ | 0.0296 (Sig.) | 0.0013 (Sig.) | Valine, leucine and isoleucine-associated metabolism |
| Valylphenylalanine | ESI+ | 1.00E-06 (Sig.) | 1.23E-06 (Sig.) | Dipeptide/amino-acid-associated metabolism |
| XMP | ESI+ | 5.55E-04 (Sig.) | 0.0195 (Sig.) | Purine metabolism |
| γ-L-Glutamyl-L-glutamic acid | ESI+ | 7.21E-15 (Sig.) | 0.0019 (Sig.) | Glutathione metabolism |

Notes. Each metabolite is listed once. D₂O and phenotype values are Benjamini-Hochberg FDR-adjusted *p*-values from the two-way ANOVA; Sig., FDR-adjusted *p* < 0.05; NS, not significant. Pathway/category assignments are not mutually exclusive. Dipeptide/amino-acid-associated and amino-acid-associated labels are biological discussion categories rather than formal database pathway assignments.

#### 1- **TCA-cycle-associated metabolites**

The responses summarised in the main text may be interpreted within the broader sensitivity of central carbon metabolism to kinetic isotope effects imposed by D₂O. Many TCA-associated enzymes involve proton- or hydride-transfer reactions that may become kinetically less favourable when deuterium replaces hydrogen. These mechanisms provide a plausible framework for interpreting the observed metabolite patterns; however, enzyme activities and metabolic fluxes were not measured directly in the present study. GDH catalyses the reversible interconversion of glutamate and α-ketoglutarate through a reaction involving hydride transfer, for which measurable kinetic isotope effects have been reported (Colen et al. 1975). D₂O could therefore influence GDH-associated reaction kinetics and contribute to changes in glutamate-associated metabolism. However, GDH activity was not measured, and NADH was not measured; therefore, the NAD⁺/NADH ratio and cellular redox state could not be determined. Moreover, significant baseline variation in glutamate abundance was detected among susceptible strains at 0% D₂O. The possible relationship between the observed glutamate patterns and GDH-associated metabolism should therefore be considered a mechanistic hypothesis rather than evidence of altered GDH activity. SDH catalyses the FAD-dependent oxidation of succinate to fumarate and transfers the resulting electrons towards ubiquinone. Proton- and electron-transfer steps associated with this process may be susceptible to kinetic isotope effects under D₂O exposure. However, succinate showed significant within-phenotype strain variation under several conditions, including both phenotype groups at 0% and 80% D₂O and tolerant isolates at 40% D₂O. The pooled succinate patterns consequently cannot be interpreted as uniform evidence of altered SDH activity across all strains. The flavin metabolite detected in the present study was flavin mononucleotide (FMN), rather than FAD. FMN participates in numerous flavin-dependent cellular processes and is not a specific cofactor of SDH. The observed FMN abundance changes are therefore interpreted more broadly as perturbation of flavin-associated metabolism and cannot be used to infer SDH activity, respiratory activity or TCA-cycle flux. Malate dehydrogenase (MDH) catalyses the reversible conversion of malate to oxaloacetate through an NAD⁺-dependent hydride-transfer reaction, which may be susceptible to kinetic isotope effects under D₂O exposure. Reduced MDH turnover could therefore shift the malate–oxaloacetate balance and contribute to altered malate abundance. However, MDH activity, metabolic flux and the NADH/NAD⁺ ratio were not measured directly. In addition, significant within-phenotype strain variation in malate abundance was detected in both susceptible and tolerant isolates at 40% and 80% D₂O. The relationship between D₂O exposure, MDH-associated metabolism and the pooled malate patterns should therefore be regarded as a mechanistic hypothesis rather than a demonstrated explanation. Taken together, these mechanistic considerations illustrate how isotope-associated changes in proton- and hydride-transfer kinetics could contribute to coordinated perturbations across linked dehydrogenase reactions. The possible interplay between measured cofactor abundance, hydrogen-transfer kinetics and electron-transport-associated processes provides a testable framework for interpreting the metabolite patterns reported in the main text. However, these mechanisms cannot be confirmed from steady-state metabolite abundances alone and require validation using enzyme-activity, redox-ratio or metabolic-flux measurements.

2- **Dipeptide and BCAA**

The D₂O-associated changes in the putatively annotated dipeptides valylphenylalanine and alanyltyrosine, together with changes in free branched-chain amino acids, may be interpreted within the context of isotope-sensitive peptide- and amino-acid-associated metabolism. Several reactions involved in peptide hydrolysis, amino-acid interconversion and downstream metabolism depend on proton- or hydride-transfer chemistry and could therefore be affected by kinetic isotope effects when deuterium replaces protium.

Peptide-derived amino acids may enter cellular metabolism through hydrolysis, uptake, transamination, deamination and subsequent carbon-skeleton metabolism. Some of these reactions involve proton transfer or depend on redox cofactors such as NAD⁺. D₂O-associated changes in reaction kinetics could therefore alter the balance between peptide-associated features, free amino acids and their downstream metabolites. However, the present data cannot distinguish between peptide uptake, synthesis, hydrolysis, transport, protein degradation or further metabolism. The partial recovery of the dipeptide features in susceptible isolates at higher D₂O concentrations should therefore not be interpreted as direct evidence that hydrolysis remained active while downstream dehydrogenase reactions became rate-limiting.

Valine, leucine and isoleucine are linked to central metabolism initially through aminotransferase-associated reactions that generate their corresponding branched-chain α-keto acids. Subsequent transformations may include proton- or hydride-transfer-dependent steps and may therefore be susceptible to isotopic substitution(Zampieri *et al.*, 2019).Such kinetic effects could contribute to changes in the relative abundances of branched-chain amino acids and related metabolites. Nevertheless, the present steady-state data do not directly demonstrate increased BCAA degradation, reduced downstream enzyme activity, impaired cofactor regeneration or altered electron-transport-chain function.

Isoleucine biosynthesis also involves the PLP-dependent enzyme threonine deaminase, which converts threonine to α-ketobutyrate through reaction chemistry involving proton transfer. This reaction could therefore be sensitive to isotopic substitution under D₂O exposure. However, threonine deaminase activity and isoleucine-biosynthetic flux were not measured, and the observed metabolite patterns cannot establish that this step was selectively inhibited.

Overall, isotope-associated effects on proton-transfer reactions, amino-acid interconversion and redox-coupled metabolism provide a plausible mechanistic framework for the dipeptide- and BCAA-associated abundance patterns reported in the main text. These mechanisms remain hypotheses because peptide origin, enzyme activity, pathway flux, cofactor regeneration and electron-transport-chain activity were not directly assessed.

3- **Cofactor, amino-acid, polyamine and glutathione-associated metabolism**

In addition to the abundance patterns described in the main text, changes in nicotinate- and nicotinamide-associated metabolites, arginine- and proline-associated metabolites, polyamines and glutathione-related compounds may be interpreted in the context of kinetic isotope effects on proton- and hydride-transfer reactions. These mechanisms provide a possible framework for interpreting the observed patterns, although enzyme activity, pathway flux and cellular redox state were not measured directly.

Within nicotinate- and nicotinamide-associated metabolism, NAD⁺, nicotinamide, nicotinate and ADP-ribose showed coordinated abundance changes. This may indicate perturbation of NAD⁺ biosynthesis, salvage, utilisation or degradation under D₂O exposure. Several reactions involved in these processes include proton-transfer or cofactor-dependent steps that could be affected by isotopic substitution. However, the relative abundance of NAD⁺ alone cannot determine NAD⁺ recycling, cofactor availability or cellular redox state because NADH was not measured and the NADH/NAD⁺ ratio could not be calculated. In addition, differences in these metabolites were already present at 0% D₂O, indicating that they may partly reflect baseline phenotype- or strain-associated variation.

The proline-associated patterns may similarly be considered in relation to isotope-sensitive reaction chemistry. In E. coli, conversion of proline to glutamate involves the PutA enzyme and occurs through sequential FAD- and NAD⁺-dependent oxidation steps. These reactions include electron- and hydride-transfer processes that could be influenced by D₂O. Changes in their kinetics could therefore contribute to altered proline abundance. Nevertheless, the present data do not demonstrate a bottleneck in proline oxidation, impaired electron transport or increased supply of peptide-derived proline. Furthermore, significant baseline variation in proline abundance was detected among susceptible strains at 0% D₂O, meaning that the pooled susceptible pattern represents an average rather than a uniform response across all three strains.

Arginine can contribute to several metabolic branches associated with glutamate, fumarate and polyamine metabolism. These pathways involve multiple enzyme-catalysed reactions that may differ in their dependence on proton transfer and redox cofactors. D₂O-associated kinetic effects could therefore alter the relative use of these branches. However, the current steady-state metabolite data cannot establish preferential pathway use, increased flux towards TCA-cycle intermediates or enhanced polyamine biosynthesis.

Polyamines such as putrescine and cadaverine have recognised roles in bacterial stress responses and in interactions with membranes and cellular macromolecules. Their observed abundance changes could arise from altered synthesis, degradation, transport or export. Although these processes may include proton-coupled enzymes and transport systems that could be influenced by D₂O, the present data cannot distinguish among these possible causes or demonstrate reduced polyamine catabolism or export.

Glutathione and γ-glutamyl-associated metabolites participate in antioxidant-associated metabolism. Proton- and hydride-transfer reactions and the availability of reducing cofactors may influence these processes under D₂O exposure. However, the observed changes do not directly demonstrate increased glutathione turnover, impaired glutathione recycling or oxidative stress. Oxidised glutathione, the GSH/GSSG ratio, NADPH/NADP⁺ and direct oxidative-stress markers were not measured. In addition, 5-oxoproline showed significant variation among susceptible strains at 80% D₂O, indicating that its pooled abundance did not represent a uniform susceptible-group response under this condition.

Overall, kinetic isotope effects on proton- and hydride-transfer reactions provide a plausible mechanistic framework for the cofactor-, amino-acid-, polyamine- and glutathione-associated abundance patterns reported in the main text. Nevertheless, differences in enzyme kinetics, cofactor regeneration, transport, electron-transport-chain activity and redox balance were not directly assessed. Their contribution to the observed phenotype-associated patterns therefore remains hypothetical and requires targeted validation.

4- **Purine and pyrimidine metabolism**

The coordinated abundance changes in IMP, XMP, GMP, AMP and related metabolites indicate perturbation of purine-associated metabolism under increasing D₂O exposure. Several reactions involved in purine synthesis, salvage, degradation and interconversion include proton-transfer, phosphotransfer or redox-dependent steps that could be influenced by kinetic isotope effects. However, decreases in steady-state nucleotide abundance do not directly demonstrate increased degradation, salvage activity or mobilisation of nucleotide pools for energy production. The observed patterns may instead reflect changes in the balance among nucleotide synthesis, salvage, degradation and cellular utilisation.

The earlier and larger decreases observed in several purine-associated metabolites in the pooled susceptible group may suggest that purine metabolism was affected differently between the phenotype groups. Nevertheless, these fold-change patterns do not establish that susceptible isolates relied more heavily on nucleotide pools for ATP or GTP production, nor do they demonstrate reduced TCA-cycle or electron-transport-chain activity. Such mechanisms remain possible explanations requiring targeted flux and energetic measurements.

The contrasting adenosine pattern in susceptible isolates may similarly reflect changes in the balance among its production, conversion, uptake, transport and utilisation. For example, altered conversion of AMP to adenosine or of adenosine to downstream purine metabolites could contribute to its accumulation. However, adenosine deaminase activity and purine-pathway flux were not measured, and the observed pattern cannot be interpreted as direct evidence of impaired adenosine turnover or cofactor limitation.

For pyrimidine-associated metabolism, changes in orotate and UMP may reflect perturbation of the balance among de novo synthesis, salvage and cellular utilisation. Pyrimidine synthesis involves multiple reactions and depends on cellular energy and precursor availability, including phosphoribosyl pyrophosphate and metabolites linked to central carbon metabolism. D₂O-associated kinetic effects on these processes could therefore contribute to the observed abundance patterns. However, ATP availability, precursor concentrations and pyrimidine-pathway flux were not measured, and the data cannot establish that PRPP became limiting or that orotate-to-UMP conversion became uncoupled.

Importantly, UMP showed significant variation among tolerant strains at 40% D₂O and within both phenotype groups at 80% D₂O. The pooled UMP patterns under these conditions should therefore be interpreted as average group-level responses rather than uniform behaviour across all strains within a phenotype.

Differences in uracil and CMP abundance may also be associated with altered pyrimidine synthesis, salvage, utilisation or nucleotide turnover. However, the present steady-state metabolite data do not directly demonstrate changes in RNA synthesis, RNA degradation, CMP processing or activation of pyrimidine-salvage pathways. The contrasting pooled patterns between susceptible and tolerant isolates therefore provide hypothesis-generating evidence of altered pyrimidine-associated metabolism rather than proof of specific RNA-turnover mechanisms.

Overall, kinetic isotope effects on proton-transfer, phosphotransfer and redox-associated reactions provide a plausible mechanistic framework for the purine- and pyrimidine-associated metabolite patterns described in the main text. However, the roles of nucleotide degradation, salvage, energy production, precursor availability and RNA turnover remain hypothetical and require targeted enzyme-activity, isotope-tracing and metabolic-flux measurements.

**Figure S2**. Overview of glycolysis and TCA cycle pathways in E. coli under D_2_O exposure, highlighting metabolic differences between resistant and sensitive isolates. Metabolites identified at MSI level 2 are shown in green; significantly altered metabolites (p < 0.05) at any D_2_O level are marked with red stars. MSI level 3 or unidentified metabolites are shown in black. Solid arrows indicate direct biochemical steps; dotted arrows represent indirect links. Pathway regions are shaded, red for TCA cycle, pale yellow for glycolysis, pale green for valine, leucine and isoleucine degradation. Box-and-whisker plots showing metabolite changes in central carbon metabolism across D_2_O conditions. Each group of six boxes is ordered from left to right: resistant 0%, sensitive 0%, resistant 40%, sensitive 40%, resistant 80%, sensitive 80%. Green indicates sensitive isolates; red indicates resistant. Darker shades reflect higher D_2_O concentrations.

**Figure S3**. Overview of glutathione, arginine and proline, nicotinate and nicotinamide metabolism pathways in E. coli under D₂O exposure, highlighting metabolic differences between resistant and sensitive isolates. Metabolites identified at MSI level 2 are shown in green; significantly altered metabolites (p < 0.05) at any D₂O level are marked with red stars. MSI level 3 or unidentified metabolites are shown in black. Solid arrows indicate direct biochemical steps; dotted arrows represent indirect links. Pathway regions are shaded, pale purple for nicotinate and nicotinamide metabolism, pale greyish blue for arginine and proline metabolism, and greyish green for glutathione metabolism. Box-and-whisker plots showing metabolite changes in mentioned pathways across D_2_O conditions. Each group of six boxes is ordered from left to right: resistant 0%, sensitive 0%, resistant 40%, sensitive 40%, resistant 80%, sensitive 80%. Green indicates sensitive isolates; red indicates resistant. Darker shades reflect higher D_2_O concentrations.

**Figure S4**. Overview of purine and pyrimidine metabolism pathways in E. coli under D₂O exposure, highlighting metabolic differences between resistant and sensitive isolates. Metabolites identified at MSI level 2 are shown in green; significantly altered metabolites (p < 0.05) at any D₂O level are marked with red stars. MSI level 3 or unidentified metabolites are shown in black. Solid arrows indicate direct biochemical steps; dotted arrows represent indirect links. Pathway regions are shaded, pale blue for purine and pale pink for pyrimidine metabolism. Box-and-whisker plots showing metabolite changes in mentioned pathways across D_2_O conditions. Each group of six boxes is ordered from left to right: resistant 0%, sensitive 0%, resistant 40%, sensitive 40%, resistant 80%, sensitive 80%. Green indicates sensitive isolates; red indicates resistant. Darker shades reflect higher D_2_O concentrations.
